# An operational approach to high resolution agro-ecological zoning in West-Africa

**DOI:** 10.1371/journal.pone.0183737

**Published:** 2017-09-05

**Authors:** Y. Le Page, Maria Vasconcelos, A. Palminha, I. Q. Melo, J. M. C. Pereira

**Affiliations:** 1 University of Lisbon, Instituto Superior de Agronomia, Centro de Estudos Florestais, Lisboa, Portugal; 2 RSeT, Remote Sensing and Environment Technology, NGO for Development, Lisboa, Portugal; International Nutrition Inc, UNITED STATES

## Abstract

The objective of this work is to develop a simple methodology for high resolution crop suitability analysis under current and future climate, easily applicable and useful in Least Developed Countries. The approach addresses both regional planning in the context of climate change projections and pre-emptive short-term rural extension interventions based on same-year agricultural season forecasts, while implemented with off-the-shelf resources. The developed tools are applied operationally in a case-study developed in three regions of Guinea-Bissau and the obtained results, as well as the advantages and limitations of methods applied, are discussed. In this paper we show how a simple approach can easily generate information on climate vulnerability and how it can be operationally used in rural extension services.

## Introduction

The natural suitability of lands for agriculture is of crucial importance in developing regions where low-input subsistence agriculture and local crop production determine food security and economic return [1]. This is especially so in Least Developed Countries (LDC) where vulnerable populations rely almost entirely on rain-fed agriculture for food and cash and are thus highly exposed to the impacts of climate and market changes [2–4]. The risks of decreased land suitability for the main staple crops, or the consequences of natural variations in yield, call for flexible strategies and institutional support to rural families so that climate resilience and adjustments of agricultural practices to local needs and conditions are facilitated [5,6].

Population growth imposes growing demands on food, feed, fiber, and bioenergy, causing difficulties in the trade-offs between different uses of land and ecosystem services [7,8]. Therefore, managing the constraints imposed by agro-ecological conditions and knowing what are the most viable crop options can facilitate planning decisions and induce choices that, while more productive, are sustainable and resilient to climatic variability [9]. However, central and regional official institutions in LDCs lack human, organization, technical, and financial resources to be able to tap into the current body of knowledge or access global data bases and tools [10]. Even though traditional knowledge can address climate risks at community scale [11–13], the full spectrum of available farming possibilities are not known and little technological means are available to support well-informed regional plans or implementation of strategies.

Several studies have shown that Agro-Ecological Zoning (AEZ) systems based on environmental variables identify the suitability for crop farming well [14,15]. An AEZ identifies areas with similar combinations of limitations and potentials for a particular crop, or land use. These areas are established according to the climatic and edaphic requirements of the specific crops, represented as a set of parameters based on elements of ecology, climatology, plant productivity, soil characteristics, and hydrology. There are various methodologies for AEZ depending on specific objectives and available data. Some approaches are focused on producing aggregated information supporting regional or national plans [16], others are specific and detailed [17–20]. Data and information about soils, land forms, climate and crops are organized and stored in databases to be incorporated into systems that implement models, pattern recognition, and optimization algorithms. Often, environmental and socioeconomic variables are georeferenced and organized in a geographic information systems (GIS) to be analysed, classified, combined, and presented in the form of maps and reports. AEZs have a wide range of potential applications, including climate change impact assessments [21,22], analysis of yield gaps [23], or explaining adaptation behaviours to climate change [24]. Therefore, it is important to develop and offer simple, effective and case-specific tools that integrate and make available the most up-to-date AEZ knowledge and produce high resolution and spatially explicit projections of current and future crop suitability.

Even though the basic concept of agro-ecological zoning is simple and supported by a mature body of knowledge, data, and tools, several reasons hamper its operational use in LDCs. The most relevant are: (1) insufficient human and technological capacity [5,25,26]; (2) complexity of the models applied; (3) coarse scale or inadequacy of outputs, which lack local specificity [9]; and (4) difficulty of directly adding newly collected local data to generate site-specific maps and predictions. Thus, it is desirable that simple, low-end but complete, reliable and richly informative instruments can be delivered to facilitate the operational use of agro-ecological information in LDCs. Such instruments can subsidize increases in food security and farming revenues under a changing climate, while minimizing the risks of unsustainability [5,27].

The work presented here stems from the following question: *Can simple knowledge-based technological instruments together with freely accessible data and software be used to support better*, *site-specific and sustainable agriculture decisions in Least Developed Countries*?

The objective of the work is thus to develop a simple and reliable methodology for high resolution crop suitability analysis under current and future climate, easily applicable and useful in LDCs. The proposed methodology addresses both regional planning purposes and pre-emptive short-term rural extension interventions based on same-year agricultural season forecasts, while implemented with off-the-shelve resources. The outputs are maps, which can be queried, overlaid and used in other cross-sector management activities to support territorial management and a landscape approach to land-use decisions.

The tools developed are applied operationally with a case-study covering three regions of Guinea-Bissau. The study included a socio-economic survey performed in May and June of 2016 in 32 communities spatially distributed in the three regions [28,29]. Information on crop productivity for AEZ validation was not collected, as farmers keep track of total production but do not know the area under cultivation, precluding the computation of crop yields. The survey results are thus not reported here, but were valuable to provide qualitative information on crop preferences, agricultural practices and production challenges.

## The case-study

### Study area

Guinea-Bissau is a small LDC in West-Africa hosting important forests and globally significant biodiversity, where economic growth and local livelihoods rely almost exclusively on natural resources and agriculture. Guinea-Bissau’s population doubled over the last 30 years, threatening ecosystem sustainability and food security. It is essential that these issues are addressed promptly as the country is highly vulnerable to climate change, with growing seasons just long enough to complete crop cycles in average climate years. Recurrent and extended droughts in West Africa over the last several decades have had substantial impacts on agricultural productivity and population vulnerability [30–32], setting an historical warning about the threat of climate change in a region relying on subsistence agriculture with little adaptation capacity.

The main rain-fed crops in Guinea-Bissau are cashew, wetland rice monoculture in cleared mangroves, and shifting upland rotations of rice, maize, millet and sorghum. The cashew sector represents nearly all of the country’s export revenue and functions as the main cash crop for local populations. Rice is the second most relevant crop and is crucial in combating poverty due to its role in food security.

Increased domestic rice production is seen as a means to both offset staple food imports and boost rural incomes (DENARPII). However, land clearing for upland rice cropping is increasingly uncontrolled and the traditional crop rotation followed by long fallows for forest regrowth is being replaced by cropping and subsequent conversion to permanent cashew plantations. This process, which induces continuous increases in land clearing with expansion of agriculture to ever more marginal lands, constitutes the main deforestation and ecosystem degradation driver. The marked loss of forest cover [33,34], compounded by projected climatic changes, greatly enhance the risk of irreversible land-productivity loss. However, there is a strong potential for growth in the agricultural sector given Guinea-Bissau’s soil and climate conditions [35].

**Fig 1:**
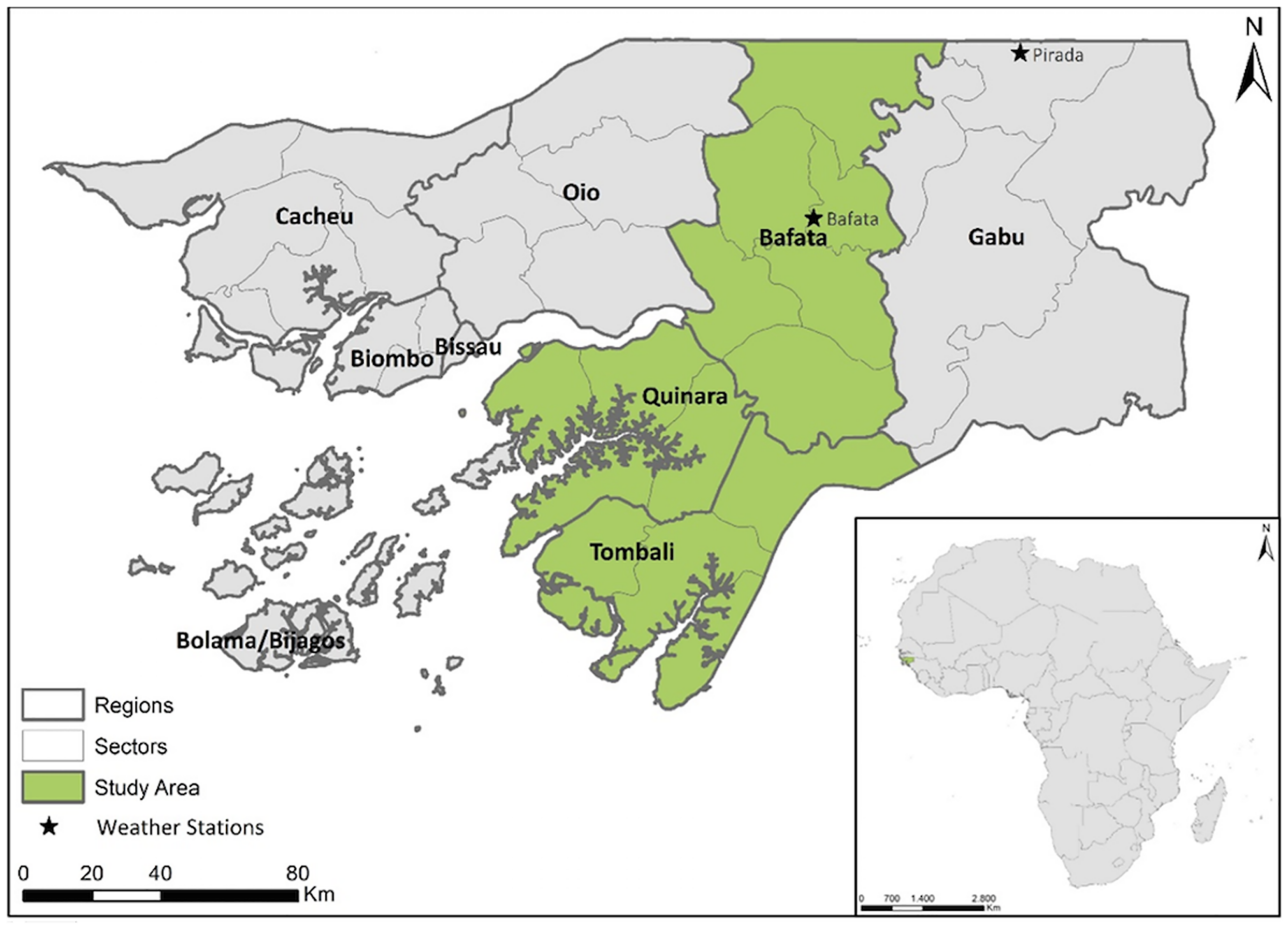
Location and delimitation of the study-area.

The study area corresponds to the three regions, shown in Fig 1, where the socioeconomic survey was performed. These three regions are representative of most agro-ecological conditions found in the national territory [36] and include all possible ethnic groups and farming systems present in the country.

### Problem description and analysis of requirements

Guinea-Bissau includes land with high agricultural potential [37] which, managed through strategic planning and informed decision making together with improvements of operational capacities, can drive national economic growth [35,38,39]. The AEZ aims at providing knowledge and tools to facilitate long-term land use decisions, such as investments in agriculture and/or conservation of wildlands, in the context of a low capacity institutional setting.

The simple comparison of mapped adequacy for crops in current conditions with that projected for future scenarios, together with information of the spatial distribution of rural communities and their size, provides important leads on location of the most vulnerable communities as expressed by potential difficulties in producing sufficient food and cash for subsistence. Additionally, AGRHYMET seasonal forecasts [40] can support the identification of same-year vulnerabilities and guide mitigation actions through rural extension services. To fulfil the requirements for planning, analysis, replicability and information display of territorial governance of the three target regions [41], all baseline data and information needed to produce the AEZ should be stored and manipulated in a geographic information system (GIS). In addition to information layers resulting from climate projections, the analysis should take into account the socioeconomic and cultural settings of the local rural communities and their spatial expression. This aspect, together with capacity building and awareness raising, is crucial for producing sufficiently participated recommendations and decisions that support realistic strategic options and plans.

## Methods

This AEZ aims at producing a set of maps which indicate and rank the suitability of each square kilometre of the study area for each selected crop in the current climatic conditions as well as in conditions projected for the future. Each specific agro-ecological map results from the combination of a climatic suitability map (current or future) with an edaphic suitability map, both produced at 1Km resolution as described below. Current and future suitability for each crop are assessed considering that no inputs are used, meaning that the effects of irrigation or fertilization, including organic–which are used to a very limited extent in Guinea-Bissau—are not accounted for.

### A- Quantification of the agro-ecological suitability

The method developed to estimate agro-ecological suitability at a given location is based on a) crop parameters, which define the optimal, sub-optimal and inadequate environmental conditions for the growth of a given crop; b) a computational assessment of a climate suitability index based on the crop parameters and on climate data; c) a computational assessment of an edaphic suitability index based on the crop parameters and on soil data; and d) the integration of the climate and edaphic suitability to infer the agro-ecological suitability and other relevant indicators.

#### A.1. Crop growth requirements

Crop growth requirements have been extensively investigated and include highly detailed, crop-specific information on the optimum combination of climate and soil conditions, on their interaction (e.g. a crop cultivated in well drained soils will need more water input), and on their evolution through the crop cycle phases (e.g. tillering, stem extension, heading, ripening). As mentioned before, the approach presented here is relatively simple and uses single variable-crop parameters, leaving aside most variables interactions and crop cycle phase considerations.

Crop growth parameters were retrieved from the FAO Ecocrop database [42], which includes a number of climatic and edaphic parameters for more than 2000 crop types, providing the flexibility aimed for while developing this methodology for its potential application to a wide range of projects. The parameters retrieved from the database can be adjusted to better represent the requirements of specific varieties or when the recent literature suggests updated values.

#### A.2. Climate suitability index

Climate suitability is computed based on a comparison of monthly climate regimes with those required for a given crop as indicated by its growth parameters (Sect. A.1.), focusing on temperature, precipitation and the length of the growing season, in two steps.

First, our approach leverages the DIVA-GIS software (version 7.5, [43]), which includes a module specifically developed to evaluate climate suitability from the Ecocrop database. For a given crop and location, DIVA-GIS first defines the ideal period for cultivation based on the monthly temperature values and calculates total precipitation during that period. The final suitability index is determined by crossing the temperature and precipitation values obtained with the growth parameters, and classified as “excellent” (index [80–100]), “very suitable” (index [60–80]), “suitable” (index [40–60]), “marginal” (index [20–40]), “very marginal” (index [0–20]) and “not suitable” (index = 0). However, defining the growing period based on temperature values is not the most adequate approach in West-Africa because the cropping period is mostly dependent on the length of the wet season.

A second component of climate suitability was thus developed to compare the length of the wet season with crop cycle durations, and implemented in the QGIS software (version 2.12, [44]). The length of the wet season is calculated as the number of consecutive months with precipitation above a given threshold, 50mm in this case study as inferred from the comparison of climate data and planting dates referred by the population and from discussions with the national agricultural planning institutions of the Bissau-Guinean Ministry of Agriculture. The difference between the length of the wet season and the crop cycle duration is then interpreted as the margin of time available for cultivation. For example, it takes about 4 months from planting to harvest for the dryland rice cultivars used in Guinea Bissau, and the wet season defined by the 50mm threshold is 5 to 6 months long in an average year from north to south of the study area. The margin for cultivation is thus +1 and +2 months, respectively. In that case, we assume that there is enough time to grow dryland rice and the climate suitability inferred from DIVA-GIS is kept unchanged.

**Fig 2:**
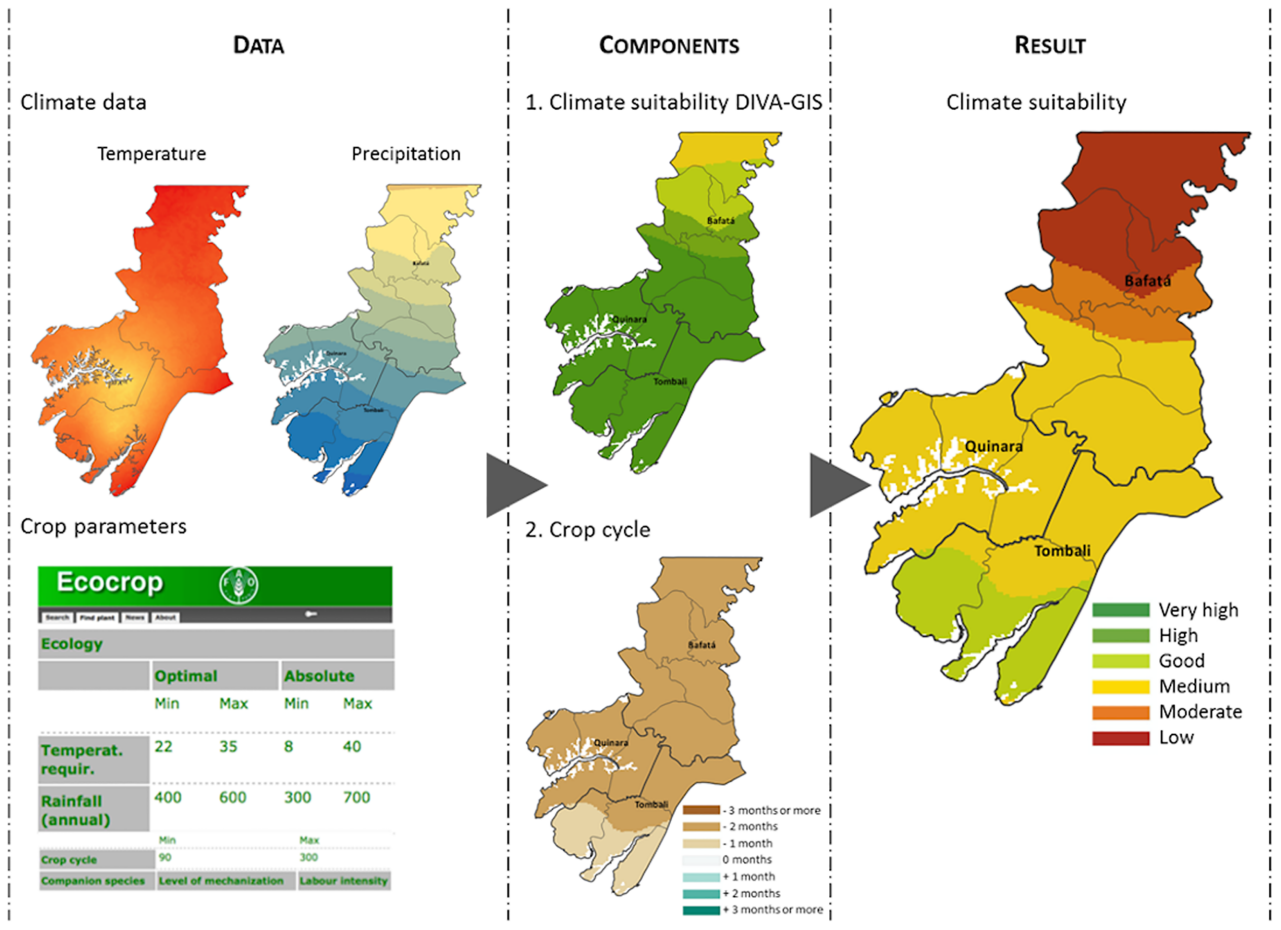
Overview of the calculation and mapping of climate suitability.

In a potential scenario of climate change where the wet season would last 2 to 3 months from north to south, however, the margin of time for cultivation would be -1 and -2 months, respectively. In that case, we decrease the suitability class of the crop by the cultivation margin: a crop with a uniform, “very suitable” DIVA-GIS climate suitability would thus be attributed the “marginal” and “suitable” final climate suitability from north to south, respectively (Fig 2). Note that this combination of the DIVA-GIS and wet season length indices is a first approach and could be applied differently.

#### A.3. Edaphic suitability

Edaphic suitability is computed based on a comparison of soil variables with the soil requirements for a given crop as indicated by its growth parameters (Sect. A.1.). For this study, we focused on soil fertility, depth to bedrock, pH, salinity and drainage, but other variables could be used.

**Fig 3:**
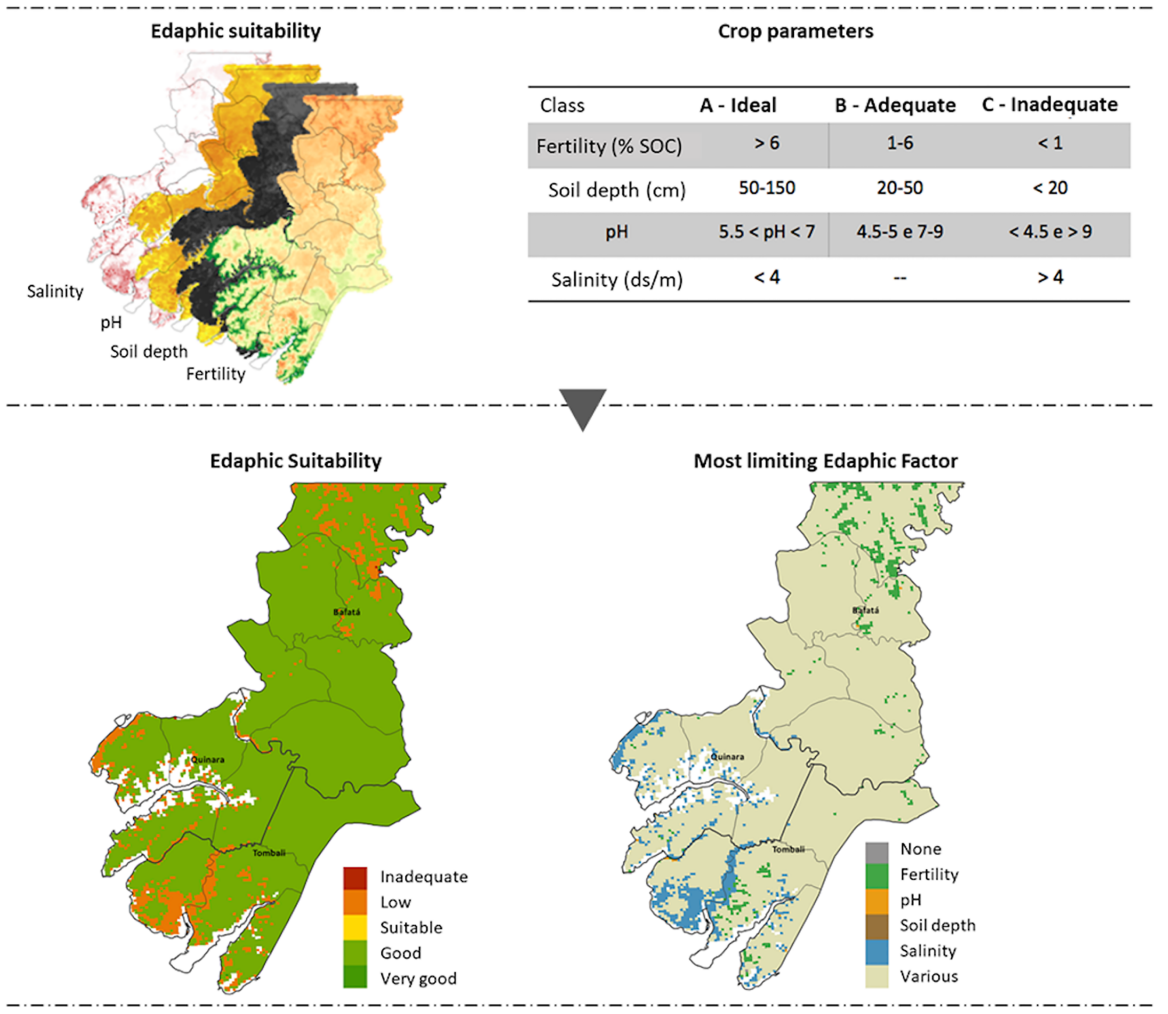
Overview of the methodology to infer the edaphic suitability and most limiting soil variables.

The edaphic suitability assessment (Fig 3) consists in the definition of three adequacy levels for each soil variables, as inferred from the Ecocrop parameters (see Sect. A.1.): “ideal”, “suitable”, and “inadequate”. For the example of dryland rice (Fig 3), a soil with a pH between 5.5 and 7 is ideal, whereas it is inadequate below 4.5 or above 9 and suitable for values in-between the suitable and inadequate classes (4.5 to 5.5 and 7 to 9). The value attributed to each class is as follow: 0 (zero) for the ideal class, 1 for the suitable class, and a number n equal to the total number of soil variables considered for the inadequate class (as explained below). The combined edaphic suitability is then computed as follows:

$$ES=\frac{100}{1+\sum_{n}ES_{n}}$$ (1)

Where ES is the combined, final edaphic suitability and ESn the suitability specific to each soil variables. This formula and the attribution of number n to the inadequate class ensures that the final ES index value discriminates situations where all variables are ideal or suitable with situations where at least one variable is inadequate and will seriously hamper crop productivity (Fig 3). Additionally, this approach enables the extraction of the most limiting soil factor(s), a feature that can support interventions such as, for example, promoting fertilizers or using cultivars with high resistance to salinity where those factors are the main issue (Fig 3).

#### A.4. Agro-ecological suitability

The combination of the Climatic Suitability and Edaphic Suitability by simple overlay and display produces the Agro-ecological suitability map for each analysed crop. The information they contain is accessed by simple queries and spatial operators in a free GIS software, Quantum GIS [44]. The agro-ecological suitability can be computed with current climate conditions as well as with climate scenarios (see Sect. B).

The application of climate scenarios enables the assessment of spatially explicit climate vulnerability for a given crop, an information that is also relevant for adaptation decision support (see Discussion). The approach applied here to compute climate vulnerability relies on a simple map algebra operation. The rationale is that the spatial distribution of the difference between current crop climatic suitability and future suitability constitutes a reliable vulnerability indicator. Indeed, populations living in an area currently unsuitable for a given crop, which is also unsuitable in a future climate situation, are less vulnerable to climate change than others currently highly dependent on this particular crop, but where decreases in suitability are expected for the future.

### B- Data

#### B.1. Climate data

We used the Worldclim data [45] to represent average precipitation and minimum, maximum and average temperature over 1950–2000 at a monthly time scale, 1km resolution and with global coverage. The data was compiled applying an interpolation procedure on data points from a number of global and regional weather station datasets, and are readily available in the format needed for the DIVA-GIS software.

**Fig 4:**
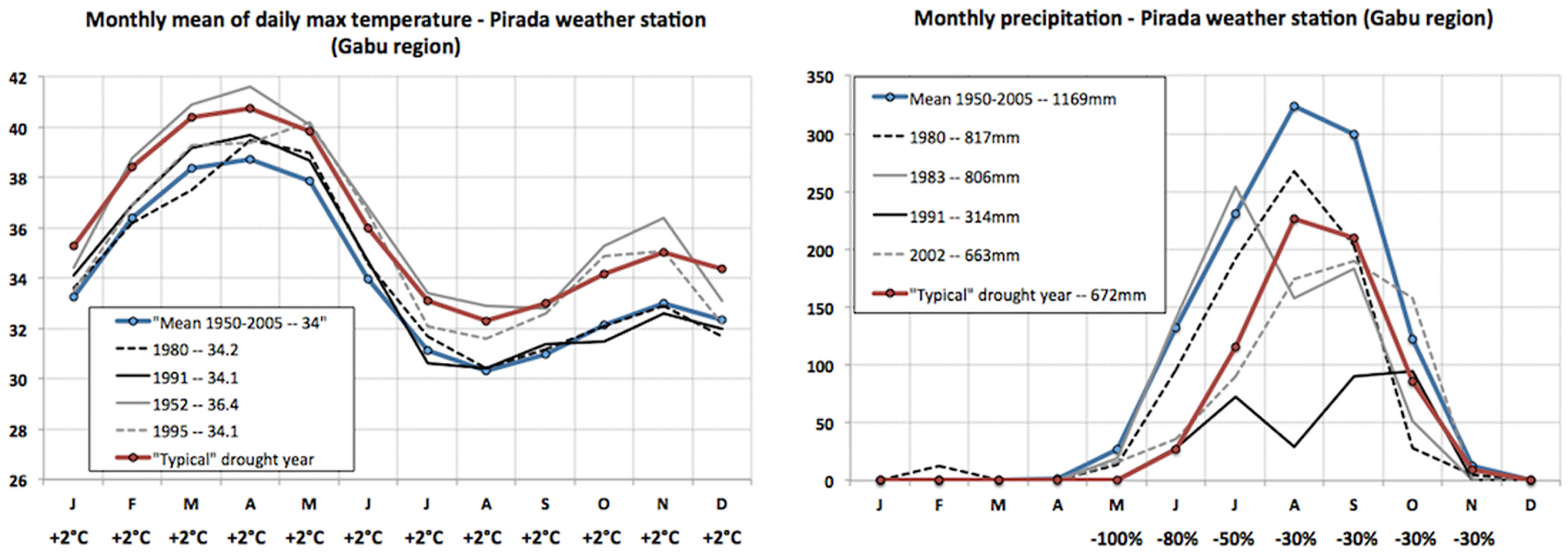
Data from the weather stations.

We also generated a set of precipitation and temperature data to represent the conditions typically observed in a dry year. These conditions were inferred based on historical drought observations from two reliable weather stations in the study region (Pirada and Bafatá, see location in Fig 1) and on the issue of a delayed wet season onset reported by the population during the field survey [28,29]. Rather than computing a statistical representation of historical droughts (e.g. average), monthly anomalies were manually adjusted to produce a simple but realistic dry year (Fig 4). In this case, annual rainfall was decreased by 42.5%, with higher percentages at the onset of the wet season (May, June and July), and temperature were increased by 2°C for all months. These dry year data can be adjusted, for example according to drought intensity expected from seasonal forecasts such as those provided by AGRHYMET.

We used two different approaches to assess the vulnerability of the agricultural sector to future climate change.

First, we explored the 2040–2060 projections from the climate models used in the Intergovernmental Panel on Climate Change Assessment Report 5 [46]. Most models project little change in Guinea Bissau, and we thus only retained the scenario and model projecting the most change–the RCP8.5 scenario from the Max-Plank Institute climate model (MPI, [47]) which projects conditions with 7–20% less precipitation and 2–3°C hotter in Guinea-Bissau. Projections from a number of the IPCC AR5 model have been applied to the Worldclim data and made available to the scientific community, facilitating the application of the agro-ecological zoning.

Changes of larger magnitude than those projected by climate models are conceivable, however, because climate models have little predictive capabilities [48–50]. Alternative scenarios can easily be implemented to consider this possibility, for example by applying a spatially/temporally uniform change to the Worldclim average conditions. A range of such “incremental” scenarios (e.g. from -50% to +50% precipitation by increments of 10%) are valuable for vulnerability assessments [51–54].

#### B.2. Soil data

We used the soil data from the World data centre for soils [55]. The ISRIC data provide a large number of physical, chemical and soil classification data at 250m resolution with global coverage, and for different soil horizons. It was derived using 158000 soil profiles and remotely sensed covariates to fit a machine learning algorithm, and the evaluation analysis established its potential for Earth-system modelling [55]. We used the 15-30cm deep horizon (except for the depth to bedrock and drainage class which have a single value per grid-cell), and the data were aggregated at the 1km resolution using the appropriate method for each variable (average fertility, pH, salinity, depth to bedrock, dominant drainage class).

### C- Crops

The crops selected for the case study are listed in Table 1. The selection was performed within a participatory process used by the EU-ACTIVA project. The consultation process consisted of several open meetings in the target regions and meetings in local communities. They included representatives from the official regional and local institutions, local NGOs, traditional community chiefs, and families of farmers.

**Table 1:** List of selected crops.

Subsistence	Cash	Emergent
Swamp rice–*Oryza Sativa*	Peanut—*Arachis hypogaea*	Cotton—*Gossypium hirsutum*
Dryland rice—*Oryza Sativa*	Banana—*Musa x paradisíaca*	Sweet potato—*Ipomoea batatas*
Bean–*Phaseolus vulgaris*	Cashew—*Anacardium occidentale*	Lemon—*Citrus limon*
Black-eyed bean—*Vigna Unguiculata*		Manioc—*Manihot esculenta*
Fundo—*Digitaria exilis*		Corn–*Zea mays*
Millet—*Pennisetum glaucum*		Palm—*Elaeis guineensis*
Sorghum—*Sorghum bicolor*		Sesame—*Sesamum indicum*

The selection aimed at including the most representative crops: subsistence crops (as expressed by the proportion of families cultivating them); cash-crops (those producing the highest revenues to the highest number of families); and emerging crops (those recently introduced or reintroduced and under experimentation by local communities). The parameters defining crop requisites are those available in Ecocrop and in the literature. An example of the parameters used in this study for dryland rice are shown in Table 2.

**Table 2:** Ecocrop parameters for dryland rice.

Class	Climatic Parameters			Edaphic Parameters			
	Temperature (°C)	Precipitation (mm)	Crop cycle (days—months)	Fertility (% organic carbon)	Soil Depth (cm)	pH	Salinity/Conductivity (dS/m)
Optimal	20–30	1500–2000	125–4	> 6	> 50	5–7	< 4
Suitable	10–20;	1000–1500;		1–6	20–50	4.5–5;	4–10
	30–38	2000–4000				7–9	
Unsuitable	< 10;	< 1000;		< 1	< 20	< 4;	> 10
	> 38	> 4000				> 9	

## Results and discussion

The developed AEZ methodology and tools are applied in Guinea-Bissau for the eighteen selected crops using current climate conditions, and conditions projected by the MPI model and by the incremental scenarios. The resulting maps are organized in QGIS, where layers with all the climate projections and soil characteristics are also stored, organized and ready to be used. Therefore, producing agro-ecologic maps for new crops requires only that the respective crop parameters be known and introduced into the alphanumeric tables of the system for processing.

**Fig 5:**
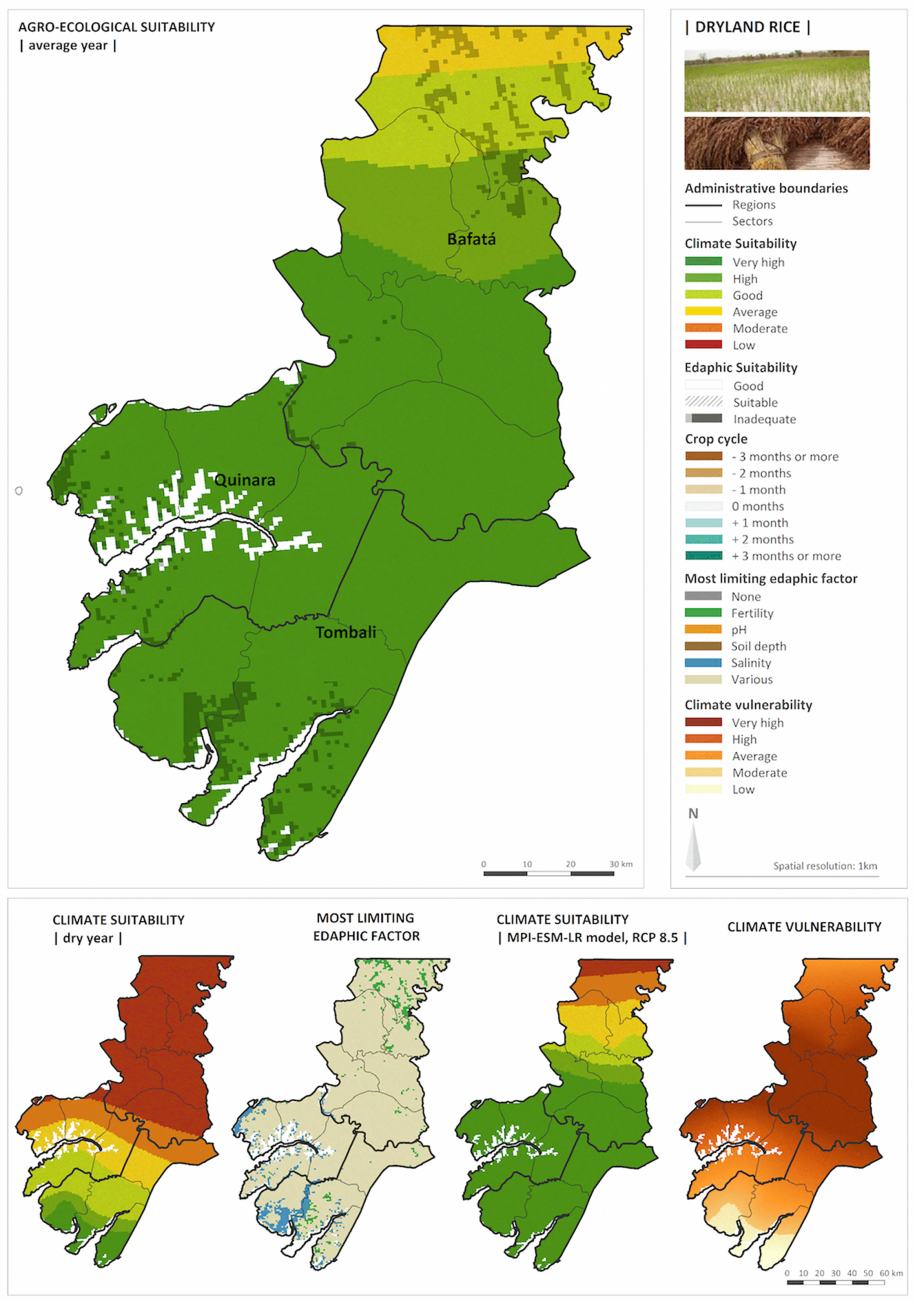
Agro-ecological results for dryland rice in the three study regions.

Fig 5 shows the AEZ for one of the most widely farmed crops in Guinea-Bissau: dryland rice, which according to the results of the socioeconomic survey is used by 54% of the rural population in the three regions. The results include the agro-ecological map produced for current climatic conditions, and a set of 4 maps illustrating the capabilities of the AEZ to identify the most limiting edaphic factors and to anticipate crop suitability in the context of seasonal forecasts and climate change projections.

**Fig 6:**
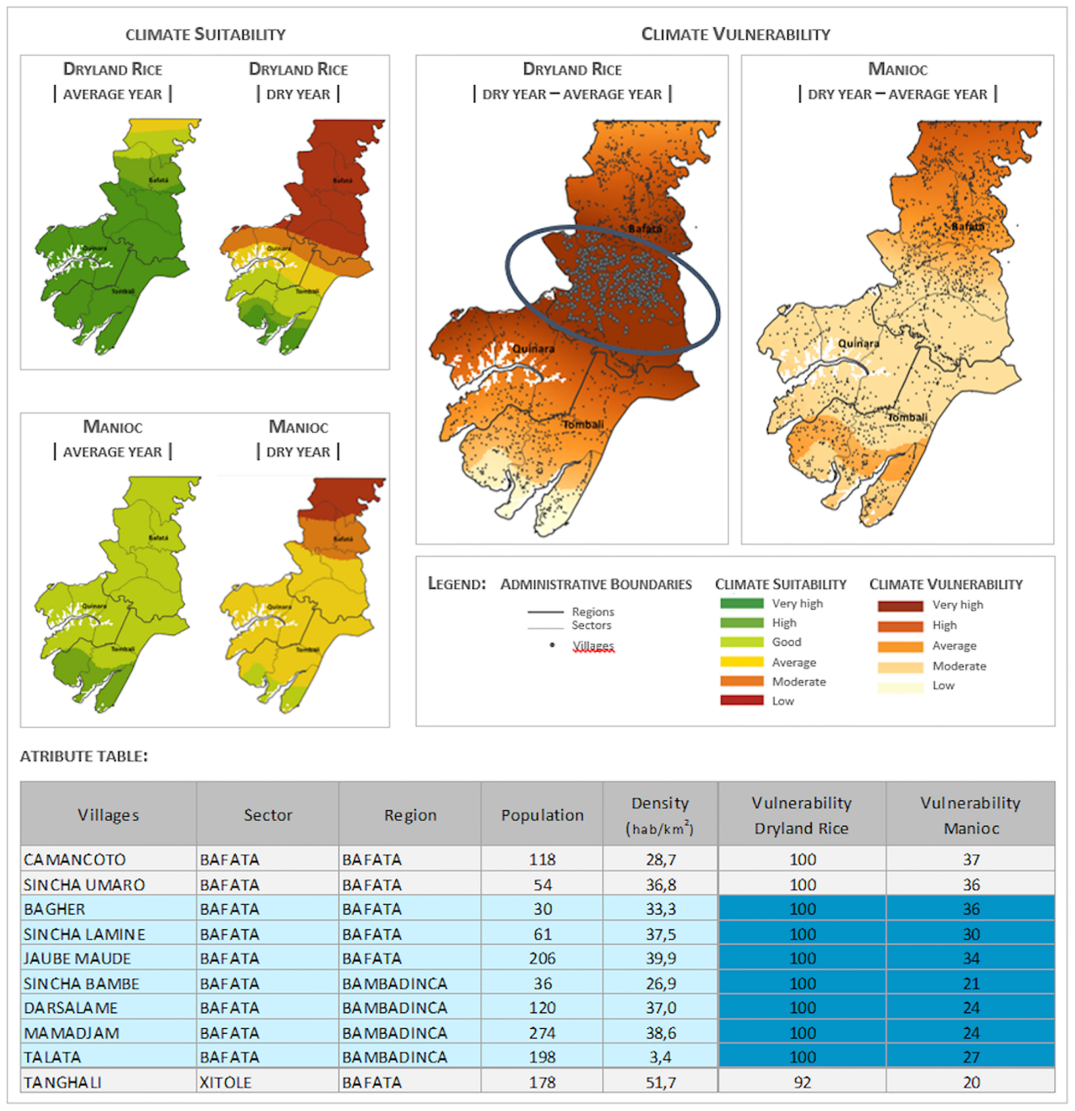
Illustration of how the information on vulnerability can be produced and utilized for rural extension services. The AEZ identifies the most vulnerable areas regarding dryland rice. Additional information included in the system, such as how many rural families are affected and their respective ethnic and age distribution characteristics, or what are acceptable alternative crops in the current year, can be used to conduct rural support activities (e.g. by locally active NGOs) such as distribution of seeds and farming advice.

Fig 6 illustrates how the AEZ can be used to support agricultural planning based on seasonal climate forecasts from AGRHYMET [40]. Every year around March, AGRHYMET provides climate forecasts for the current agricultural season, which starts with the first plantings by the end of May or beginning of June, when the rains begin. The climate data for an average year can then be altered accordingly, for example to represent a typical dry year as done in this study (see Sect. B.1.). The AEZ presented here can then provide suitability maps for all the parameterized crops in its data base. Since census data and the results of the socioeconomic survey are also available in the system, the relative importance of dryland rice for the sustainability of each local community is also available. The potential impact of the forecast can thus be anticipated to support adaptation (e.g. provide information about crops which might be particularly affected; identify alternative crops better adapted to droughts; seeds distribution programs).

The system proposed in this study is designed to be both a stand-alone tool and a component of a broader land use management system structured to implement a landscape approach [56] to mainstreaming increases in agricultural output in the context of sustainable development strategies. It is an effective, low-cost and flexible tool, which can be deployed to guide public policies and programs in agricultural development. A one-week capacity building workshop was delivered to the staff of the Ministry of Agriculture in Guinea-Bissau. The workshop was sufficient to capacitate the technical staff with the key concepts and capabilities of the AEZ system, but 2–3 week sessions are ideally required to achieve full capacitation. The developed AEZ system could also support agricultural research and region-wide programs designed to explore coherent and compatible international procedures responding to global challenges.

Several aspects of the AEZ system would however benefit from further development to improve its usefulness and accessibility. First, a thorough evaluation of the suitability maps is needed at a range of spatial scales to provide guidance on their applicability from local projects to national or international programs. In countries lacking the necessary data on agricultural productivity–as in the case of Guinea Bissau–a visual evaluation of the main spatial patterns by local institutions and specialists is essential. Second, the computation of the climate suitability index in two separate steps (overall climate suitability with DIVA-GIS and crop cycle with QGIS) is cumbersome. The implementation of the whole AEZ system within a single computational platform (e.g. as a QGIS plugin) would make it more consistent and ease the transfer of knowledge through training. Finally, this AEZ system focuses on climate and edaphic suitability as one component for guidance, but other factors affecting agricultural productivity–such as pests—need to be considered in parallel.

Particularly relevant for future applications of the AEZ system is the support of local institutions. The information gathered during the Participative Rural Appraisal were key to each phase of the project, including the evaluation of soil/climate input data, the adjustment of crop parameters, and the analysis of the suitability maps. To achieve relevant support for decision making, the AEZ system should thus be deployed in collaboration with institutions that have extensive knowledge of the agro-ecological characteristics of the study area.

## Conclusions

This study demonstrates how a simple approach can generate relevant information on climate vulnerability and how it can be operationally used in rural extension services. In spite of current opportunities and international commitments in LDCs, governance difficulties, compounded by lack of knowledge, information, and sustainable financing still prevent true transformative processes towards integrated and spatially explicit agricultural development. The work performed and the results obtained highlight the opportunity and need of developing tailored software that can quickly, easily, and inexpensively grant access to the full body of agro-ecological knowledge and data to assist food production and environmental sustainability. This study is the first application of this new AEZ approach and should be complemented by additional research and developments to improve its performance and support potential.

## Supporting information

S1 File Crop parameters and agro-ecological zoning for all crops studied in the project. (DOCX)
