# Supplementary material for: An operational approach to high resolution agro-ecological zoning in West-Africa
Source: PLoS One. 2017 Sep 5;12(9):e0183737. doi: 10.1371/journal.pone.0183737 (PMC5584804; doi:10.1371/journal.pone.0183737)
Supplement: S1 File — (DOCX) [file pone.0183737.s001.docx]

**An operational approach to high resolution agro-ecological zoning in West-Africa**

Y. Le Page^1¶^*, Maria Vasconcelos^1¶^, A. Palminha^1,2&^, I.Q. Melo^1,2&^, JMC Pereira^1^

**Supplementary information**

**Crop parameters and agro-ecological zoning for all crops studied in the project**

Below are the suitability parameters and agro-ecological zonings for the crops included in the Guinea-Bissau case study. They were produced as part of the EU-funded ACTIVA project (Integrated territorial and collective actions to develop agriculture for Guinea-Bissau). In this operational context, some Ecocrop parameters were adjusted in concertation with local institutions.

**Index:**

1. Subsistence crops 2

2. Cash crops 8

3. Emergent crops 11

# Subsistence crops

**
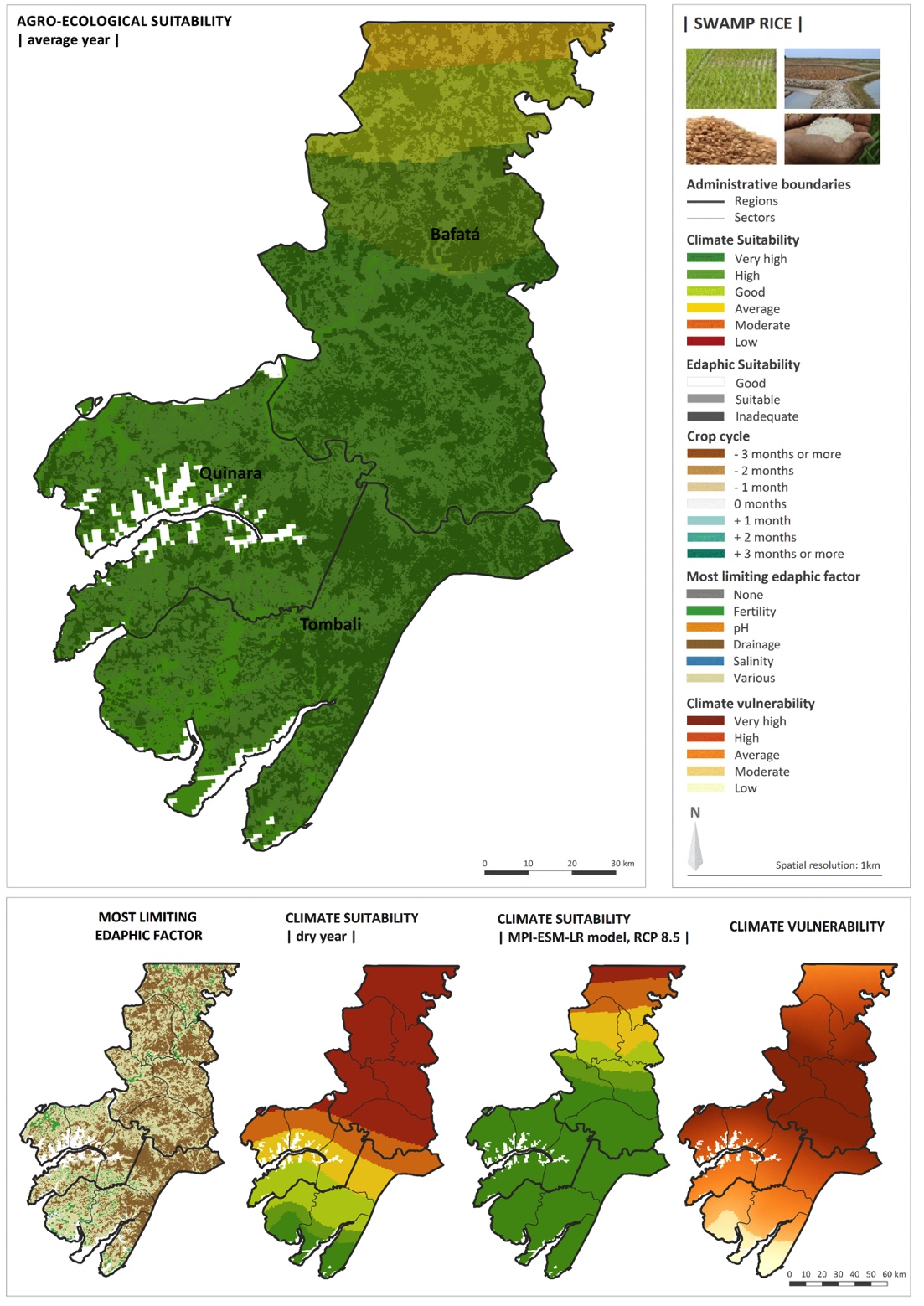
**

| **Class** | **Climatic Parameters** | | | **Edaphic Parameters** | | | |
| --- | --- | --- | --- | --- | --- | --- | --- |
|  | **Temperature (ºC)** | **Precipitation (mm)** | **Crop cycle (days – months)** | **Fertility (% organic carbon)** | **drainage** | **pH** | **Salinity/**  **Conductiv-ity (dS/m)** |
| Optimal | 20 – 30 | 1500 – 2000 | 125 - 4 | > 6 | very low | 5 – 7 | < 4 |
| Suitable | 10 – 20;  30 – 38 | 1000 – 1500;  2000 – 4000 |  | 1 – 6 | low | 4.5 – 5;  7 – 9 | 4 – 10 |
| Unsuitable | < 10 ; > 38 | < 1000 ; > 4000 |  | < 1 | other | < 4 ; > 9 | > 10 |

**
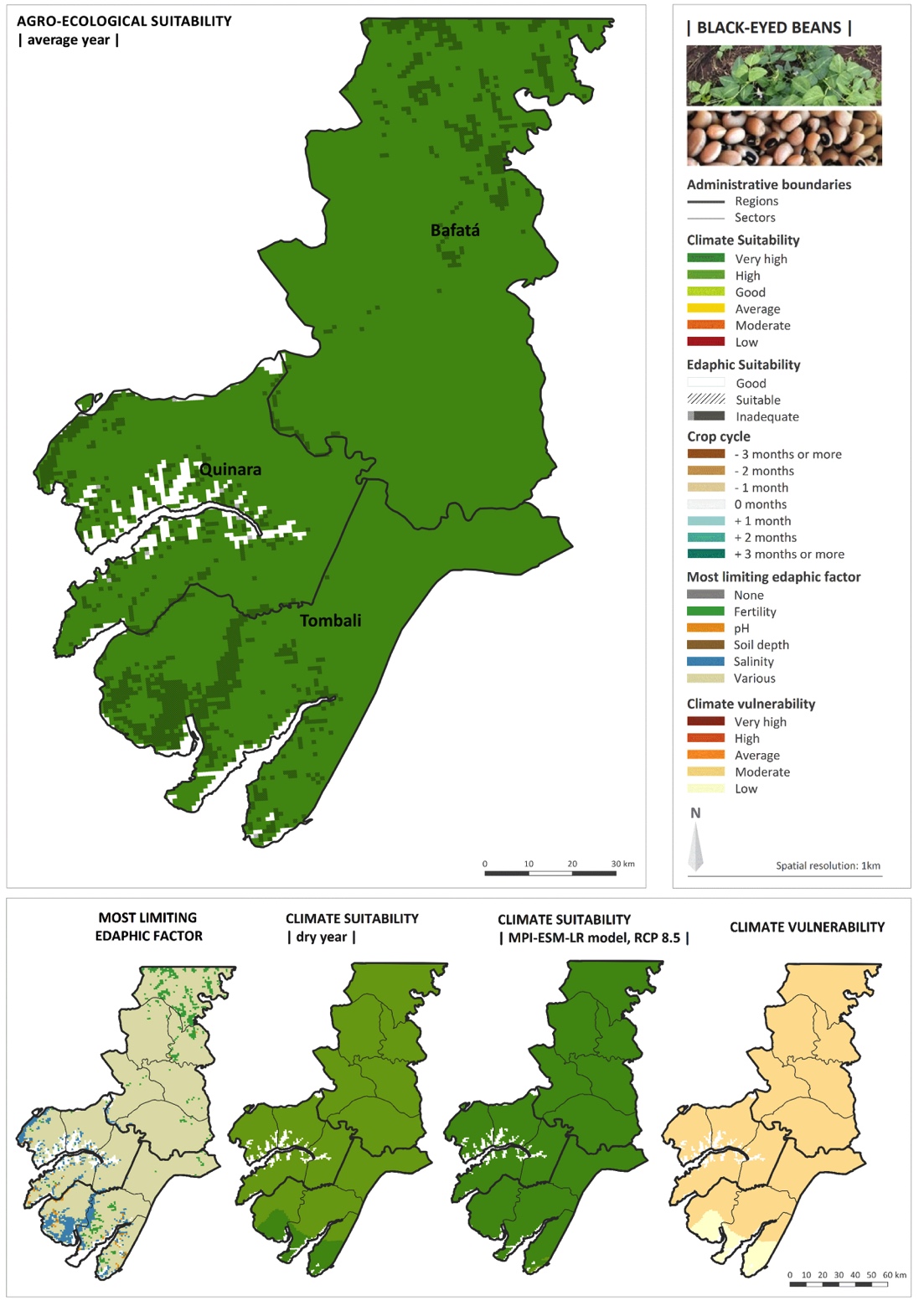
**

| **Class** | **Climatic Parameters** | | | **Edaphic Parameters** | | | |
| --- | --- | --- | --- | --- | --- | --- | --- |
|  | **Temperature (ºC)** | **Precipitation (mm)** | **Crop cycle (days – months)** | **Fertility (% organic carbon)** | **Soil Depth (cm)** | **pH** | **Salinity/**  **Conductiv-ity (dS/m)** |
| Optimal | 15 – 30 | 300 – 1000 | 45 - 2 | > 4 | > 50 | 5.5 – 7.5 | < 4 |
| Suitable | 5 – 15;  30 – 36 | 200 – 300;  1000 – 2000 |  | 1 – 4 | 20 - 50 | 4.3 – 5.5;  7.5 – 8.8 | 4 – 10 |
| Unsuitable | < 5 ; > 36 | < 200 ; > 2000 |  | < 1 | < 20 | < 4.3 ;  > 8.8 | > 10 |

**
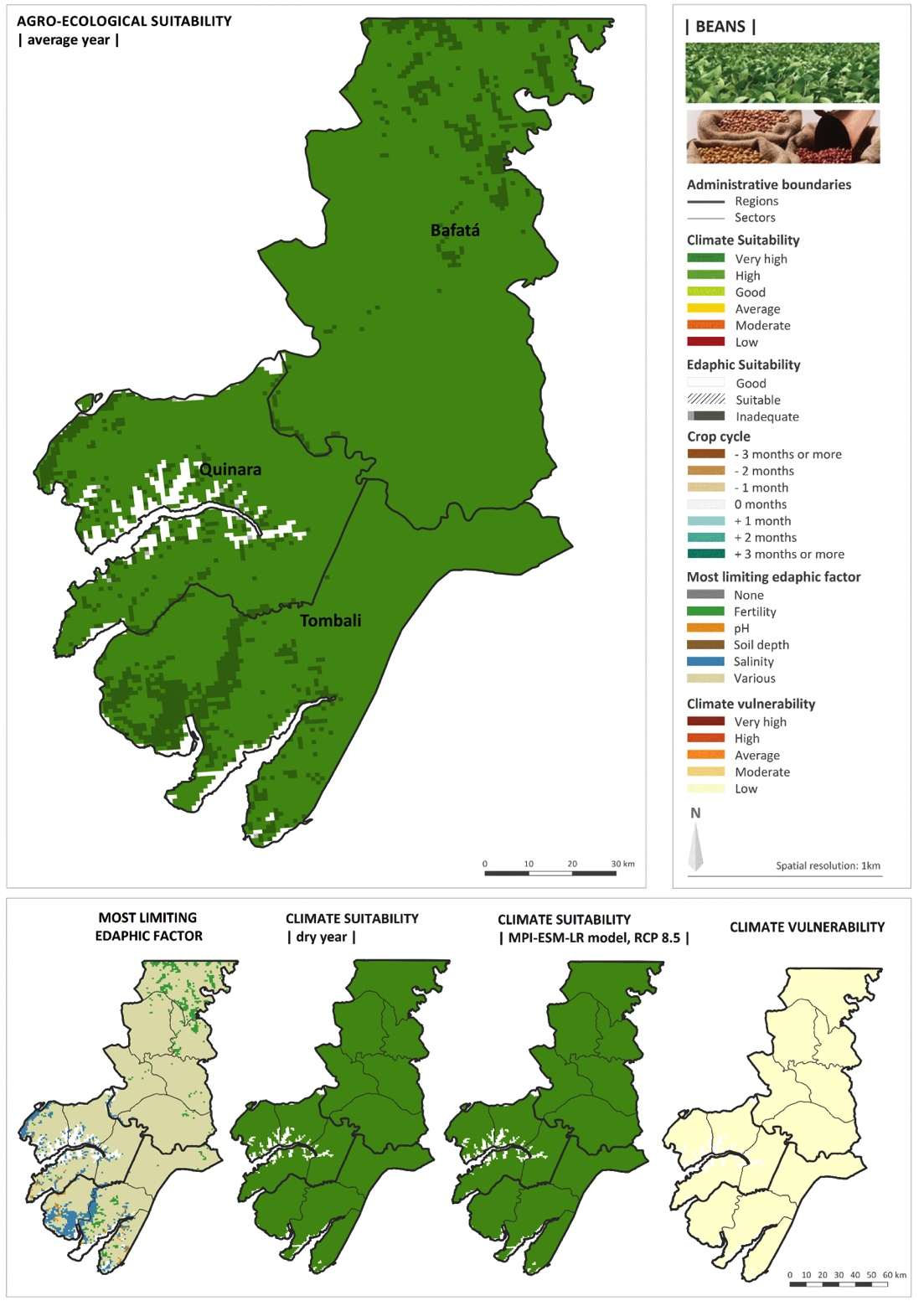
**

| **Class** | **Climatic Parameters** | | | **Edaphic Parameters** | | | |
| --- | --- | --- | --- | --- | --- | --- | --- |
|  | **Temperature (ºC)** | **Precipitation (mm)** | **Crop cycle (days – months)** | **Fertility (% organic carbon)** | **Soil Depth (cm)** | **pH** | **Salinity/**  **Conductiv-ity (dS/m)** |
| Optimal | 15 – 30 | 600 – 1500 | 120 - 4 | > 4 | > 50 | 5.5 – 7.5 | < 4 |
| Suitable | 5 – 15;  30 – 36 | 400 – 600;  1500 – 2000 |  | 1 – 4 | 20 - 50 | 4.3 – 5.5;  7.5 – 8.8 | - |
| Unsuitable | < 5 ; > 36 | < 400 ; > 2000 |  | < 1 | < 20 | < 4.3 ;  > 8.8 | > 4 |

**
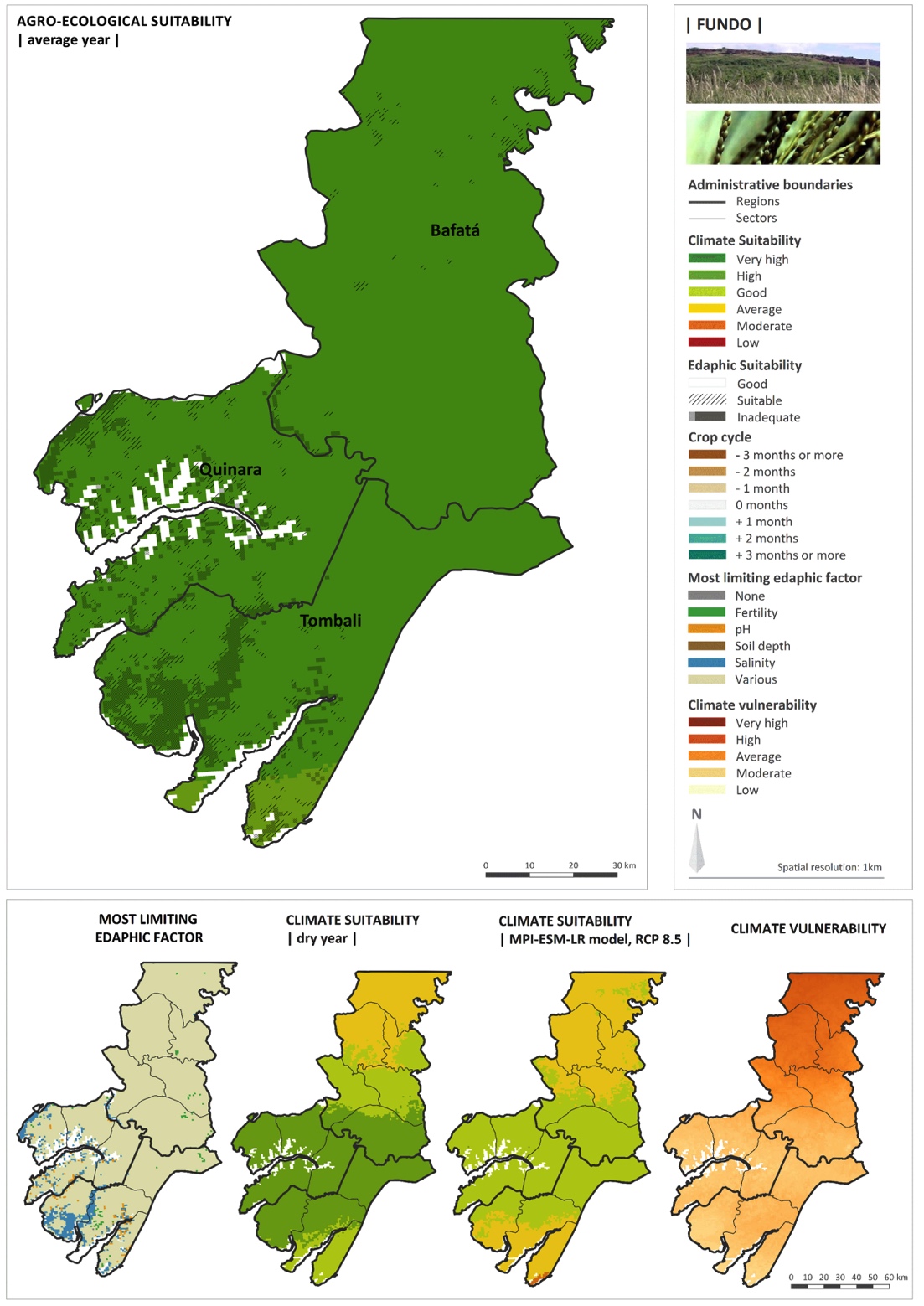
**

| **Class** | **Climatic Parameters** | | | **Edaphic Parameters** | | | |
| --- | --- | --- | --- | --- | --- | --- | --- |
|  | **Temperature (ºC)** | **Precipitation (mm)** | **Crop cycle (days – months)** | **Fertility (% organic carbon)** | **Soil Depth (cm)** | **pH** | **Salinity/**  **Conductiv-ity (dS/m)** |
| Optimal | 18 – 27 | 900 – 1600 | 100 - 3 | > 3 | > 50 | 5.5 – 6.5 | < 2 |
| Suitable | 5 – 18;  27 – 31 | 400 – 900;  1600 – 2800 |  | 0.5 – 3 | 20 - 50 | 4.5 – 5.5;  6.5 – 7.1 | 2 - 4 |
| Unsuitable | < 5 ; > 31 | < 400 ; > 2800 |  | < 0.5 | < 20 | < 4.5 ;  > 7.1 | > 4 |

**
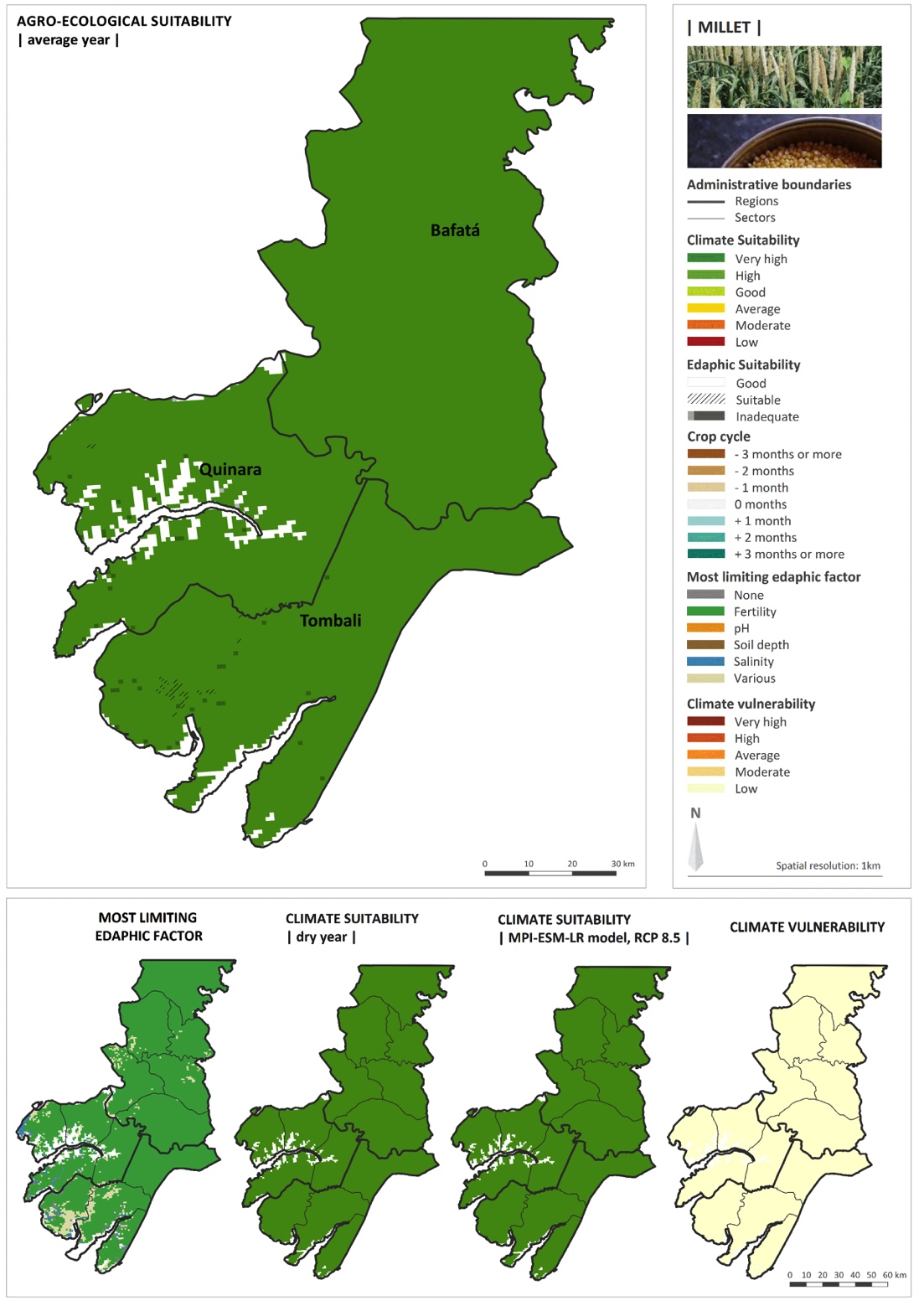
**

| **Class** | **Climatic Parameters** | | | **Edaphic Parameters** | | | |
| --- | --- | --- | --- | --- | --- | --- | --- |
|  | **Temperature (ºC)** | **Precipitation (mm)** | **Crop cycle (days – months)** | **Fertility (% organic carbon)** | **Soil Depth (cm)** | **pH** | **Salinity/**  **Conductiv-ity (dS/m)** |
| Optimal | 25 – 35 | 400 – 900 | 90 - 3 | > 4 | > 50 | 5 – 6.5 | < 4 |
| Suitable | 12 – 25;  35 – 40 | 200 – 400;  900 – 1700 |  | 0.5 – 4 | 20 - 50 | 4 – 5;  6.5 – 8.3 | 4 – 10 |
| Unsuitable | < 12 ; > 40 | < 200 ; > 1700 |  | < 0.5 | < 20 | < 4 ;  > 8.3 | > 10 |

**
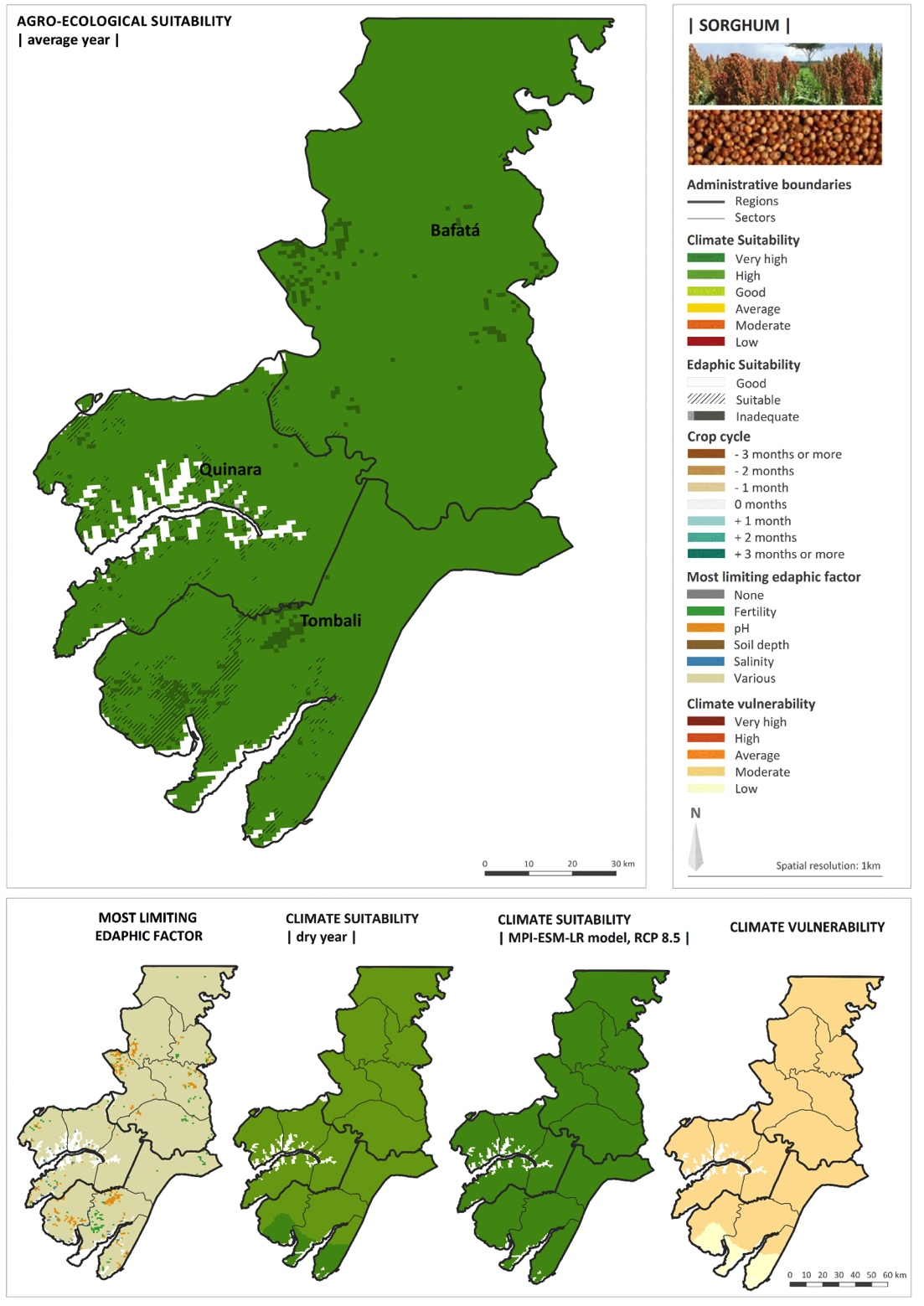
**

| **Class** | **Climatic Parameters** | | | **Edaphic Parameters** | | | |
| --- | --- | --- | --- | --- | --- | --- | --- |
|  | **Temperature (ºC)** | **Precipitation (mm)** | **Crop cycle (days – months)** | **Fertility (% organic carbon)** | **Soil Depth (cm)** | **pH** | **Salinity/**  **Conductiv-ity (dS/m)** |
| Optimal | 24 – 35 | 500 – 1000 | 120 - 4 | > 4 | > 50 | 5.5 – 7.5 | < 4 |
| Suitable | 10 – 24;  35 – 50 | 300 – 500;  1000 – 2000 |  | 0.5 – 4 | 20 - 50 | 5 – 5.5;  7.5 – 8 | 4 – 10 |
| Unsuitable | < 10 ; > 50 | < 300 ; > 2000 |  | < 0.5 | < 20 | < 5 ;  > 8 | > 10 |

# Cash crops

**
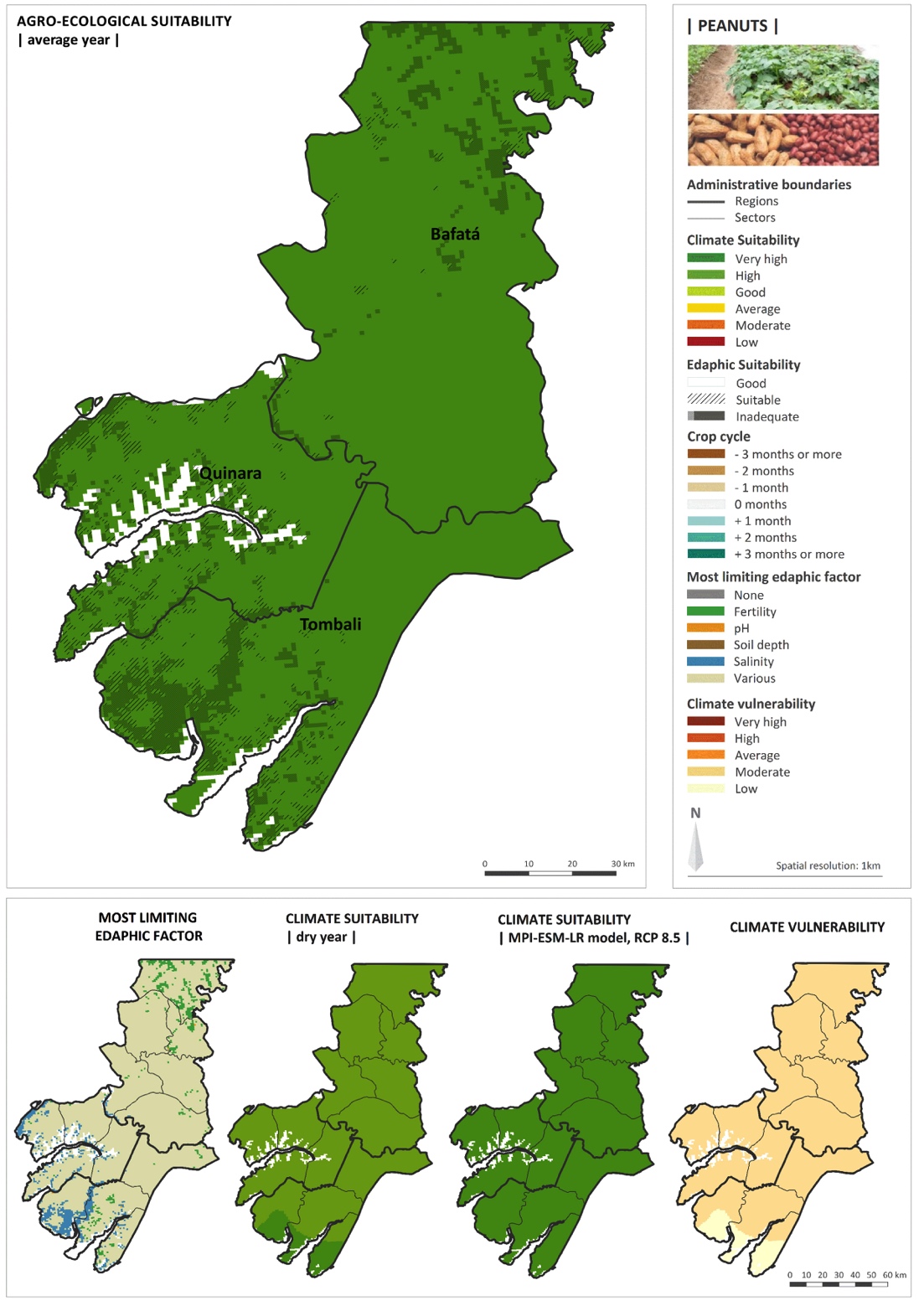
**

| **Class** | **Climatic Parameters** | | | **Edaphic Parameters** | | | |
| --- | --- | --- | --- | --- | --- | --- | --- |
|  | **Temperature (ºC)** | **Precipitation (mm)** | **Crop cycle (days – months)** | **Fertility (% organic carbon)** | **Soil Depth (cm)** | **pH** | **Salinity/**  **Conductiv-ity (dS/m)** |
| Optimal | 22 – 32 | 600 – 1500 | 110 - 4 | > 5 | > 50 | 5.5 – 6.5 | < 2 |
| Suitable | 10 – 22;  32 – 45 | 400 – 600;  1500 – 4000 |  | 1 – 5 | 20 - 50 | 4.5 – 5.5;  6.5 – 8.5 | 2 – 4 |
| Unsuitable | < 10 ; > 45 | < 400 ; > 4000 |  | < 1 | < 20 | < 4.5 ;  > 8.5 | > 4 |

**
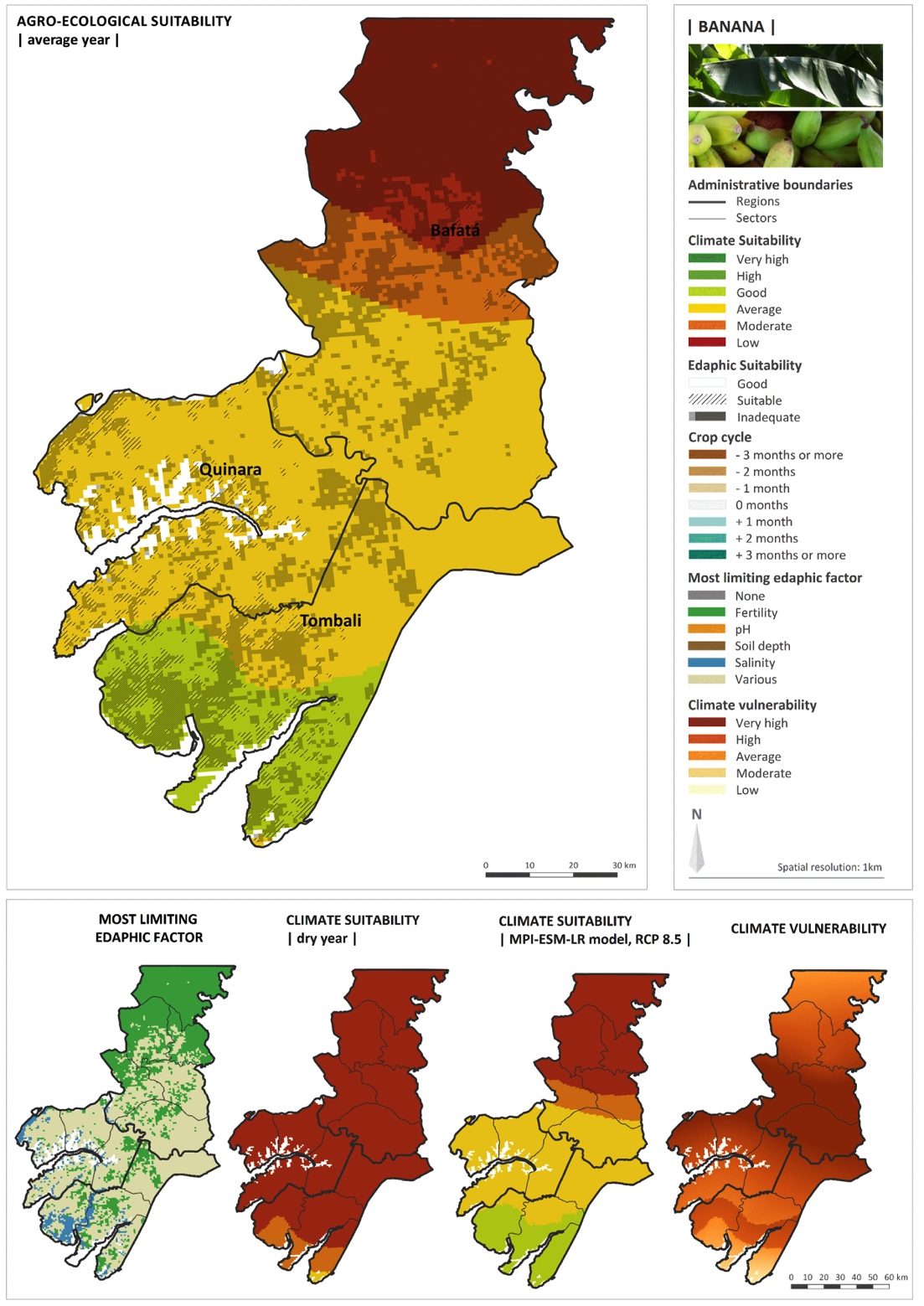
**

| **Class** | **Climatic Parameters** | | | **Edaphic Parameters** | | | |
| --- | --- | --- | --- | --- | --- | --- | --- |
|  | **Temperature (ºC)** | **Precipitation (mm)** | **Crop cycle (days – months)** | **Fertility (% organic carbon)** | **Soil Depth (cm)** | **pH** | **Salinity/**  **Conductiv-ity (dS/m)** |
| Optimal | 18 – 32 | 1700 – 2500 | 210 - 7 | > 6 | > 150 | 5 – 7 | < 2 |
| Suitable | 10 – 18;  32 – 38 | 1000 – 1700;  2500 – 3500 |  | 1.5 – 6 | 50 - 150 | 4.5 – 5;  7 – 7.5 | 2 – 4 |
| Unsuitable | < 10 ; > 38 | < 1000 ; > 3500 |  | < 1.5 | < 50 | < 4.5 ;  > 7.5 | > 4 |

**
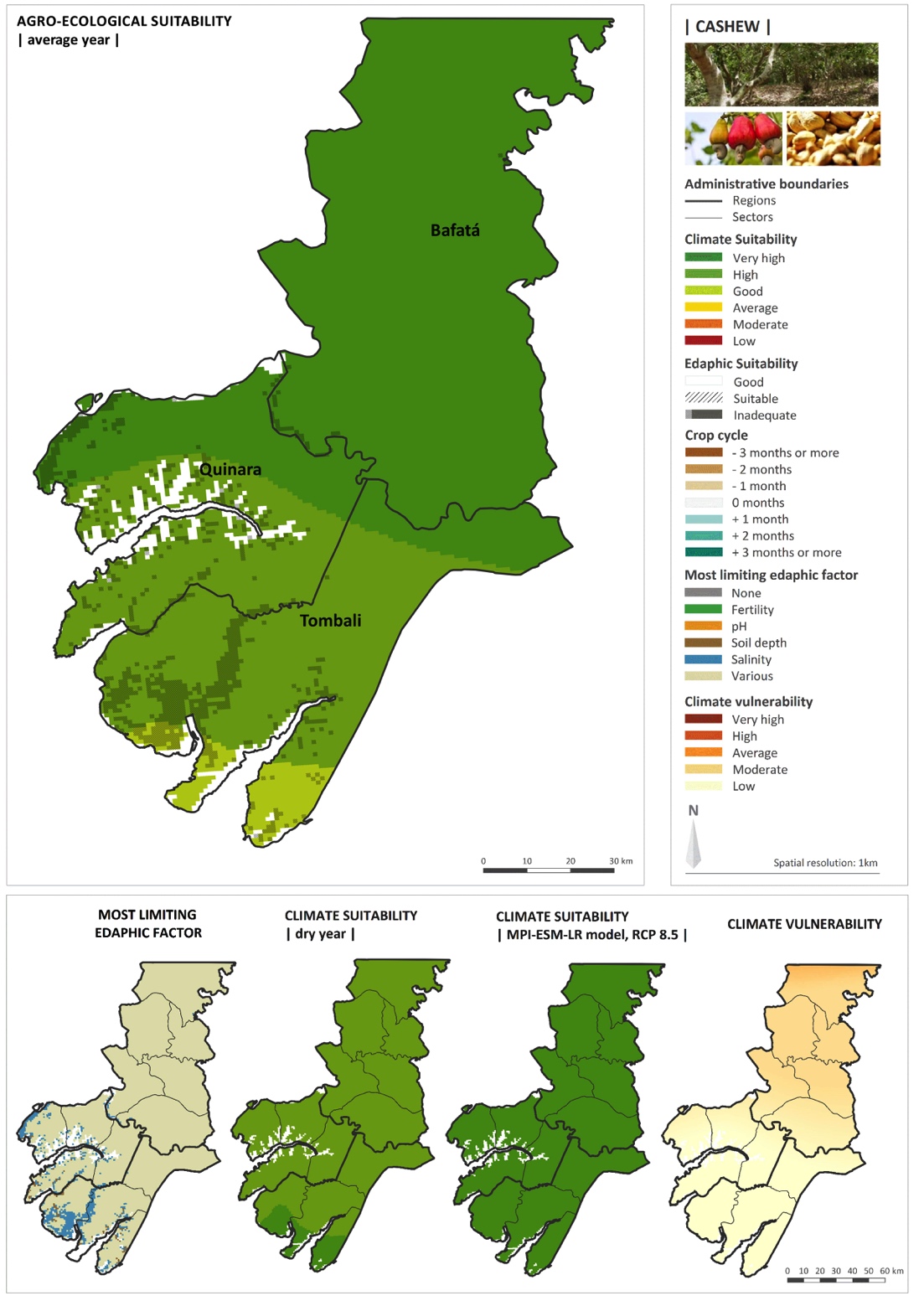
**

| **Class** | **Climatic Parameters** | | | **Edaphic Parameters** | | | |
| --- | --- | --- | --- | --- | --- | --- | --- |
|  | **Temperature (ºC)** | **Precipitation (mm)** | **Crop cycle (days – months)** | **Fertility (% organic carbon)** | **Soil Depth (cm)** | **pH** | **Salinity/**  **Conductiv-ity (dS/m)** |
| Optimal | 15 – 35 | 750 – 1600 | 120 - 4 | > 4 | > 150 | 4.5 – 6.5 | < 4 |
| Suitable | 5 – 15;  35 – 46 | 400 – 750;  1600 – 4000 |  | 0.5 – 4 | 50 - 150 | 3.8 – 4.5;  6.5 – 8.7 | – |
| Unsuitable | < 5 ; > 46 | < 400 ; > 4000 |  | < 0.5 | < 50 | < 3.8 ;  > 8.7 | > 4 |

# Emergent crops

**
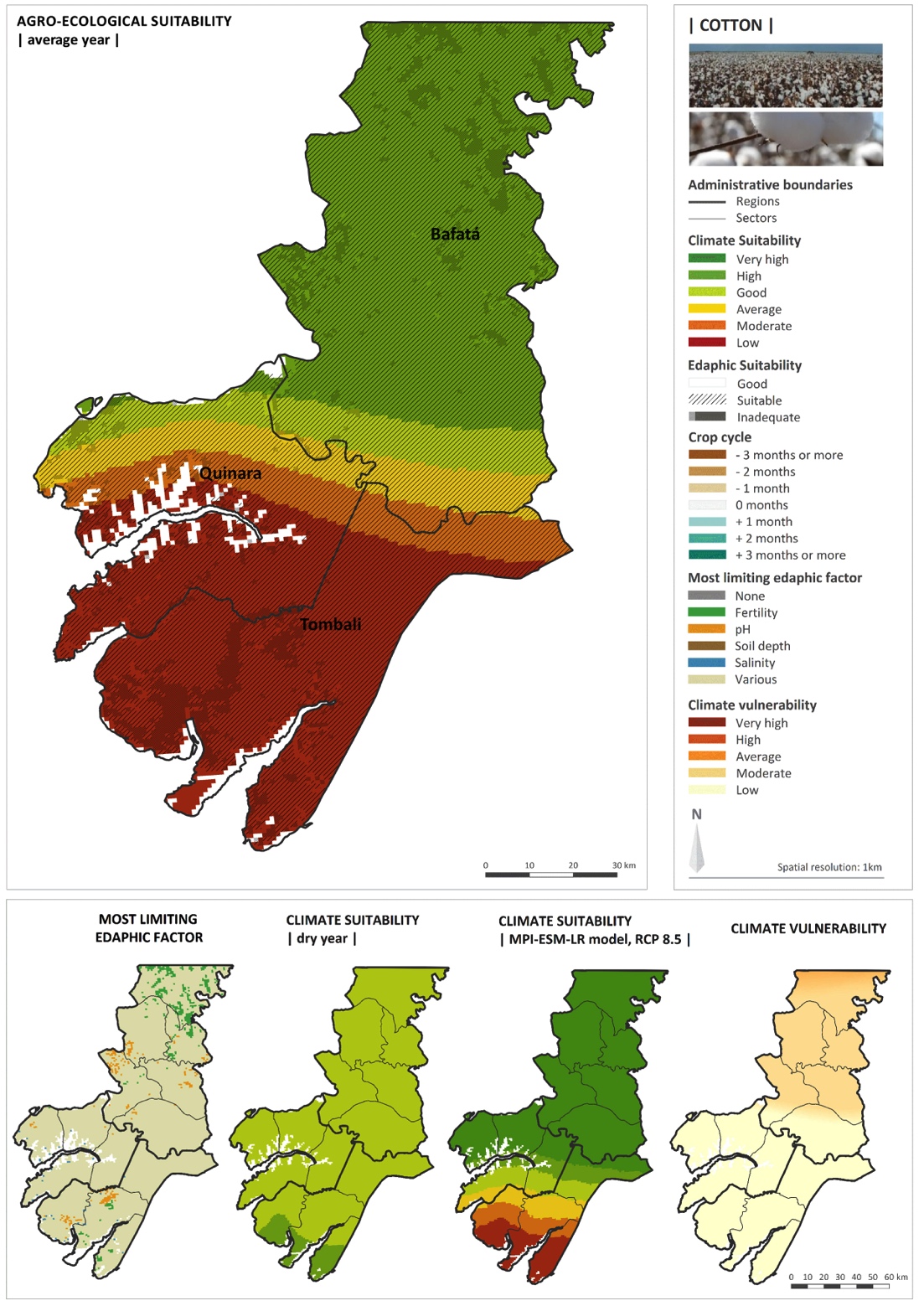
**

| **Class** | **Climatic Parameters** | | | **Edaphic Parameters** | | | |
| --- | --- | --- | --- | --- | --- | --- | --- |
|  | **Temperature (ºC)** | **Precipitation (mm)** | **Crop cycle (days – months)** | **Fertility (% organic carbon)** | **Soil Depth (cm)** | **pH** | **Salinity/**  **Conductiv-ity (dS/m)** |
| Optimal | 22 – 36 | 750 – 1200 | 175 – 6 | > 4 | > 150 | 6 – 8 | < 4 |
| Suitable | 15 – 22;  36 – 42 | 450 – 750;  1200 – 1500 |  | 1 – 4 | 50 - 150 | 5 – 6;  8 – 9.5 | 4 – 10 |
| Unsuitable | < 15 ; > 42 | < 450 ; > 1500 |  | < 1 | < 50 | < 5 ;  > 9.5 | > 10 |

**
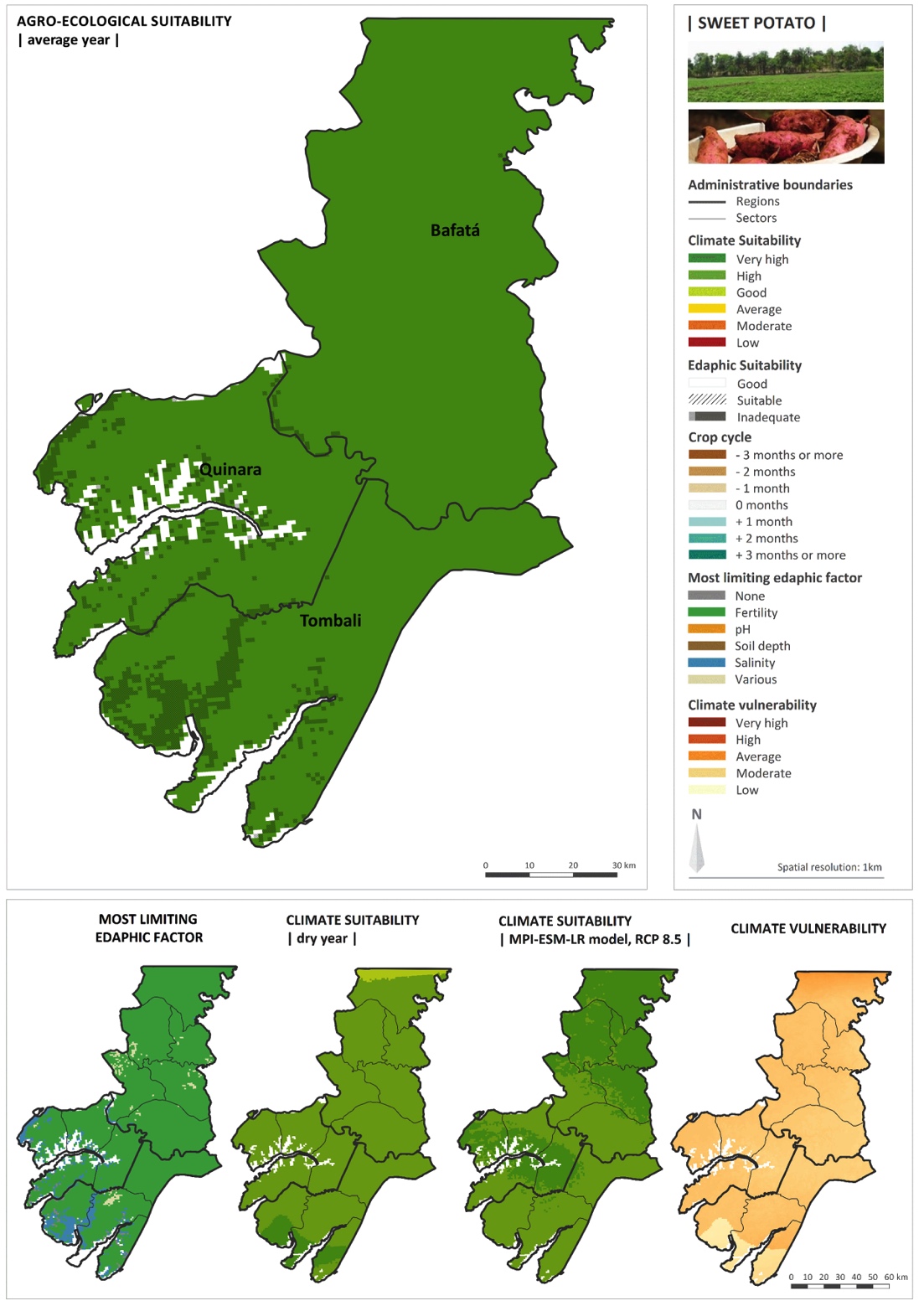
**

| **Class** | **Climatic Parameters** | | | **Edaphic Parameters** | | | |
| --- | --- | --- | --- | --- | --- | --- | --- |
|  | **Temperature (ºC)** | **Precipitation (mm)** | **Crop cycle (days – months)** | **Fertility (% organic carbon)** | **Soil Depth (cm)** | **pH** | **Salinity/**  **Conductiv-ity (dS/m)** |
| Optimal | 18 – 28 | 750 – 1500 | 120 - 4 | > 4 | > 50 | 5 – 7 | < 4 |
| Suitable | 10 – 18;  28 – 38 | 400 – 750;  1500 – 5000 |  | 0.5 – 4 | 20 - 50 | 4 – 5;  7 – 8 | – |
| Unsuitable | < 10 ; > 38 | < 400 ; > 5000 |  | < 0.5 | < 20 | < 4 ;  > 8 | > 4 |

**
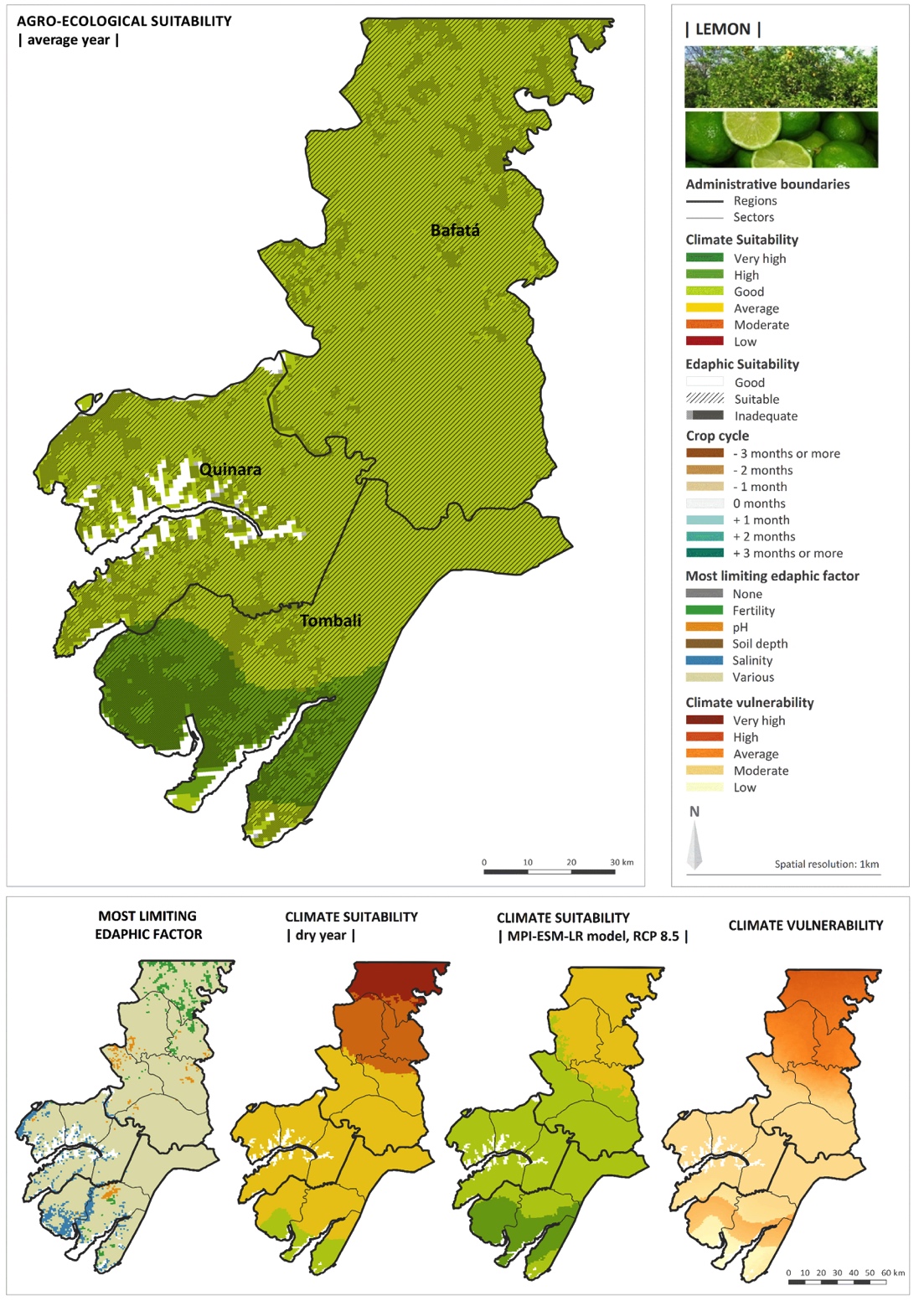
**

| **Class** | **Climatic Parameters** | | | **Edaphic Parameters** | | | |
| --- | --- | --- | --- | --- | --- | --- | --- |
|  | **Temperature (ºC)** | **Precipitation (mm)** | **Crop cycle (days – months)** | **Fertility (% organic carbon)** | **Soil Depth (cm)** | **pH** | **Salinity/**  **Conductiv-ity (dS/m)** |
| Optimal | 17 – 30 | 1000 – 2300 | 180 - 6 | > 5 | > 150 | 6 – 7 | < 2 |
| Suitable | 12 – 17;  30 – 36 | 300 – 1000;  2300 – 4000 |  | 1 – 5 | 50 - 150 | 5 – 6;  7 – 8 | 2 – 4 |
| Unsuitable | < 12 ; > 36 | < 300 ; > 4000 |  | < 1 | < 50 | < 5 ;  > 8 | > 4 |

**
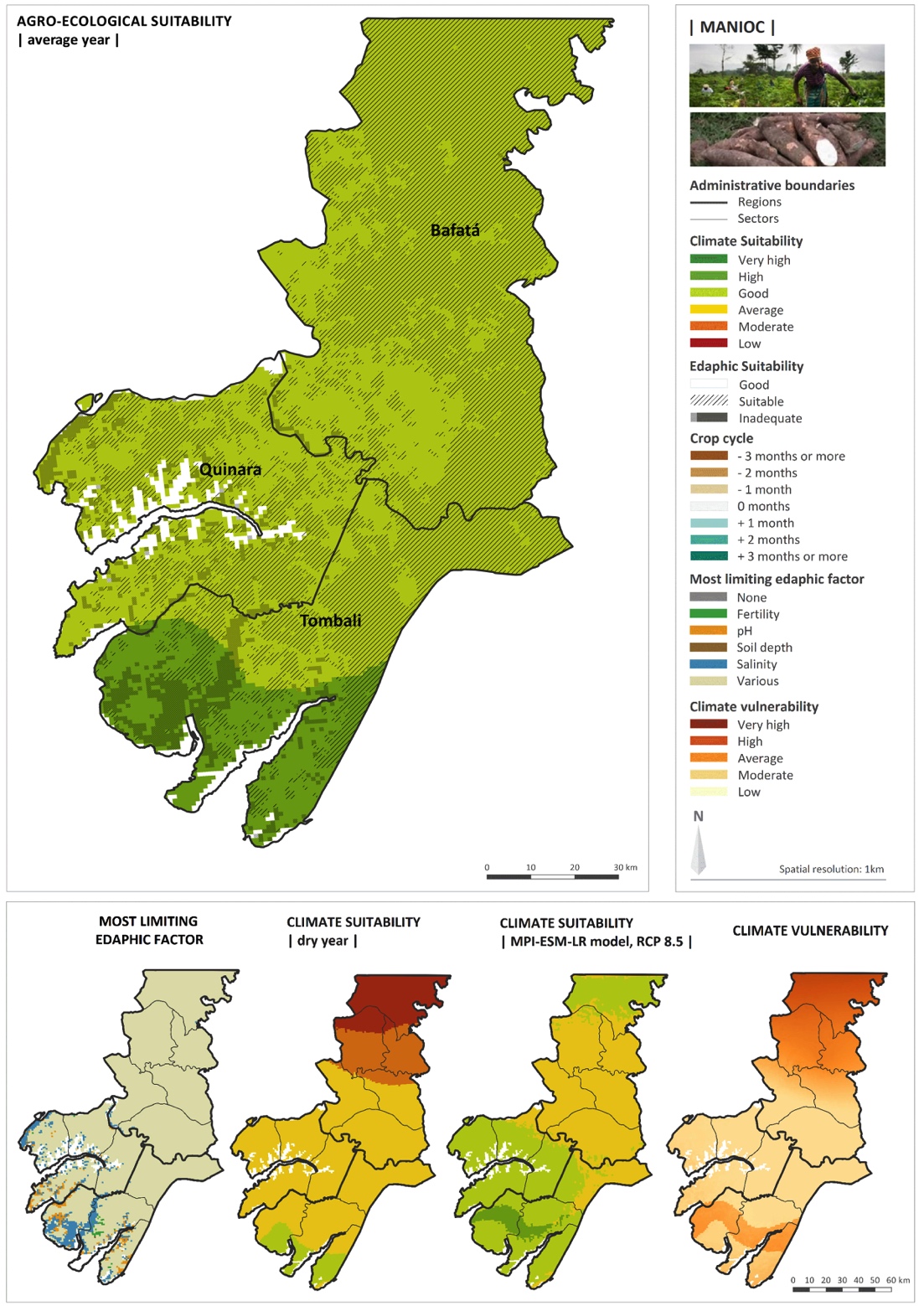
**

| **Class** | **Climatic Parameters** | | | **Edaphic Parameters** | | | |
| --- | --- | --- | --- | --- | --- | --- | --- |
|  | **Temperature (ºC)** | **Precipitation (mm)** | **Crop cycle (days – months)** | **Fertility (% organic carbon)** | **Soil Depth (cm)** | **pH** | **Salinity/**  **Conductiv-ity (dS/m)** |
| Optimal | 20 – 29 | 1000 – 1500 | 200 - 6 | > 3 | > 100 | 5.5 – 8 | < 4 |
| Suitable | 10 – 20;  29 – 35 | 500 – 1000;  1500 – 5000 |  | 0.5 – 3 | 50 - 100 | 4 – 5.5;  8 – 9 | – |
| Unsuitable | < 10 ; > 35 | < 500 ; > 5000 |  | < 0.5 | < 50 | < 4 ;  > 9 | > 4 |

**
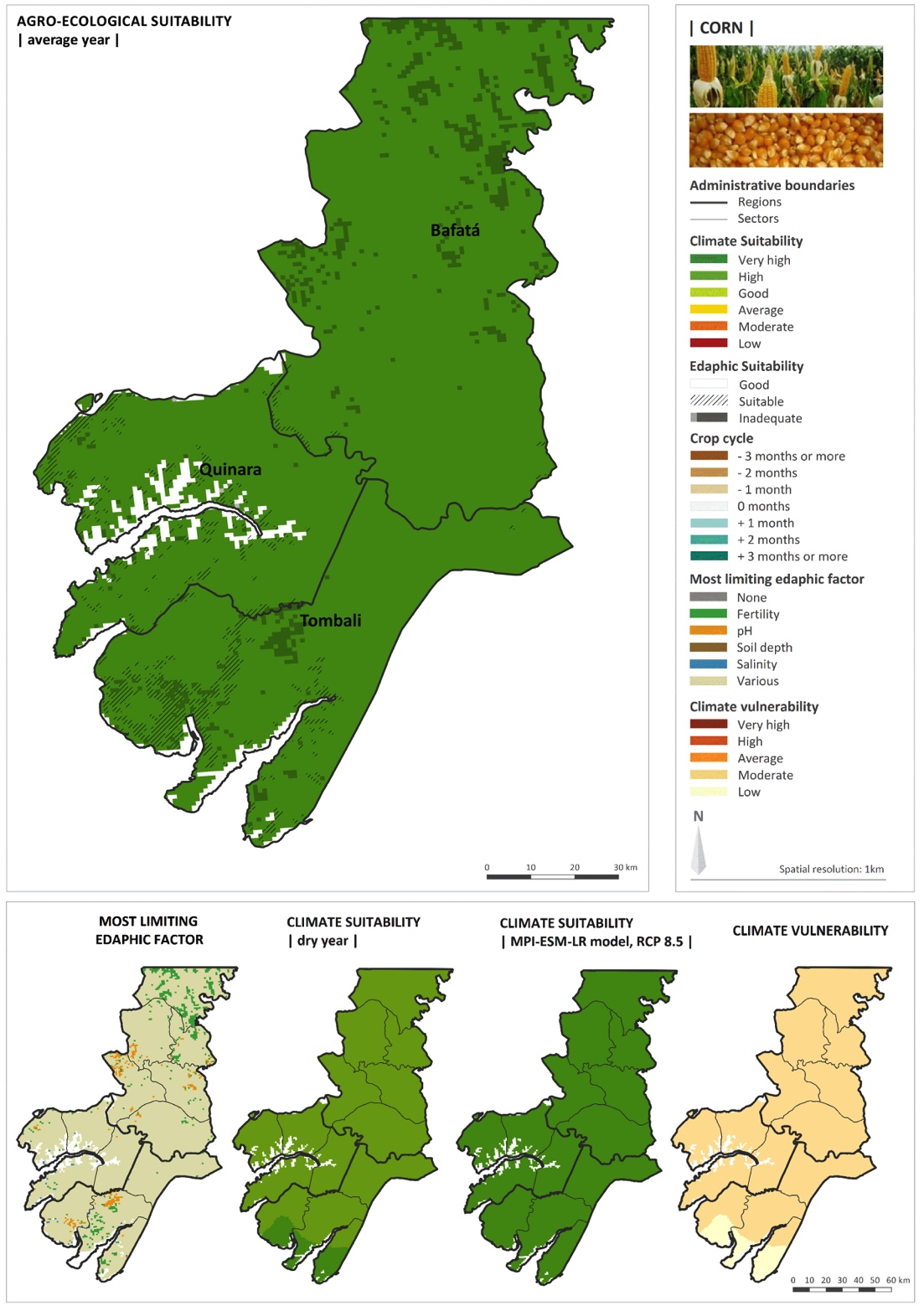
**

| **Class** | **Climatic Parameters** | | | **Edaphic Parameters** | | | |
| --- | --- | --- | --- | --- | --- | --- | --- |
|  | **Temperature (ºC)** | **Precipitation (mm)** | **Crop cycle (days – months)** | **Fertility (% organic carbon)** | **Soil Depth (cm)** | **pH** | **Salinity/**  **Conductiv-ity (dS/m)** |
| Optimal | 18 – 33 | 600 – 1200 | 120 - 4 | > 6 | > 60 | 5.5 – 7 | < 4 |
| Suitable | 10 – 18;  33 – 47 | 400 – 600;  1200 – 1800 |  | 1 – 6 | 30 – 60 | 5 – 5.5;  7 – 8.5 | 4 – 10 |
| Unsuitable | < 10 ; > 47 | < 400 ; > 1800 |  | < 1 | < 30 | < 5 ;  > 8.5 | > 10 |

**
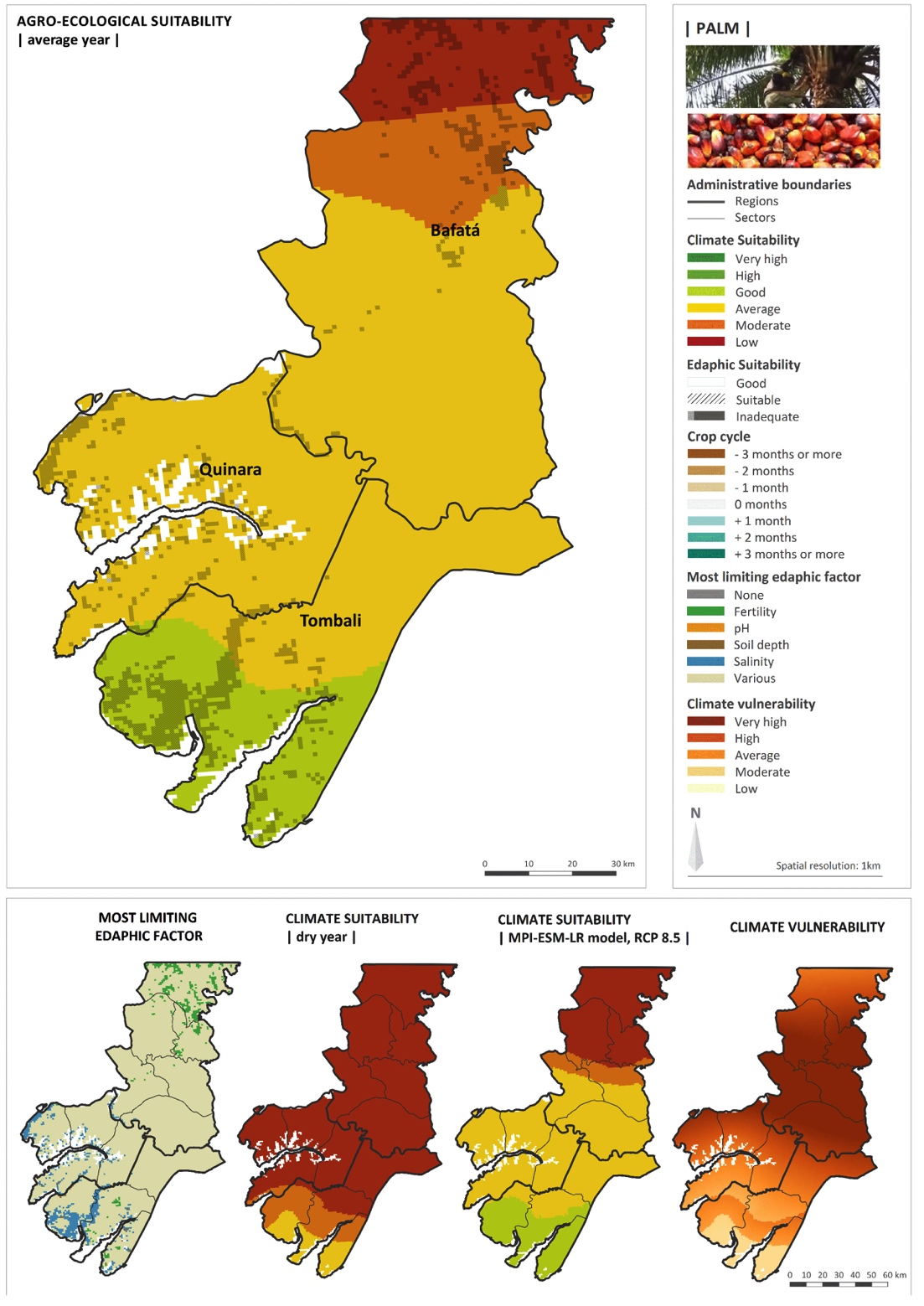
**

| **Class** | **Climatic Parameters** | | | **Edaphic Parameters** | | | |
| --- | --- | --- | --- | --- | --- | --- | --- |
|  | **Temperature (ºC)** | **Precipitation (mm)** | **Crop cycle (days – months)** | **Fertility (% organic carbon)** | **Soil Depth (cm)** | **pH** | **Salinity/**  **Conductiv-ity (dS/m)** |
| Optimal | 20 – 35 | 1500 – 3000 | 210 - 7 | > 5 | > 150 | 4.5 – 6 | < 4 |
| Suitable | 12 – 20;  35 – 38 | 1000 – 1500;  3000 – 8000 |  | 1 – 5 | 50 - 150 | 3.2 – 4.5;  6 – 8 | – |
| Unsuitable | < 12 ; > 38 | < 1000 ; > 8000 |  | < 1 | < 50 | < 3.2 ;  > 8 | > 4 |

**
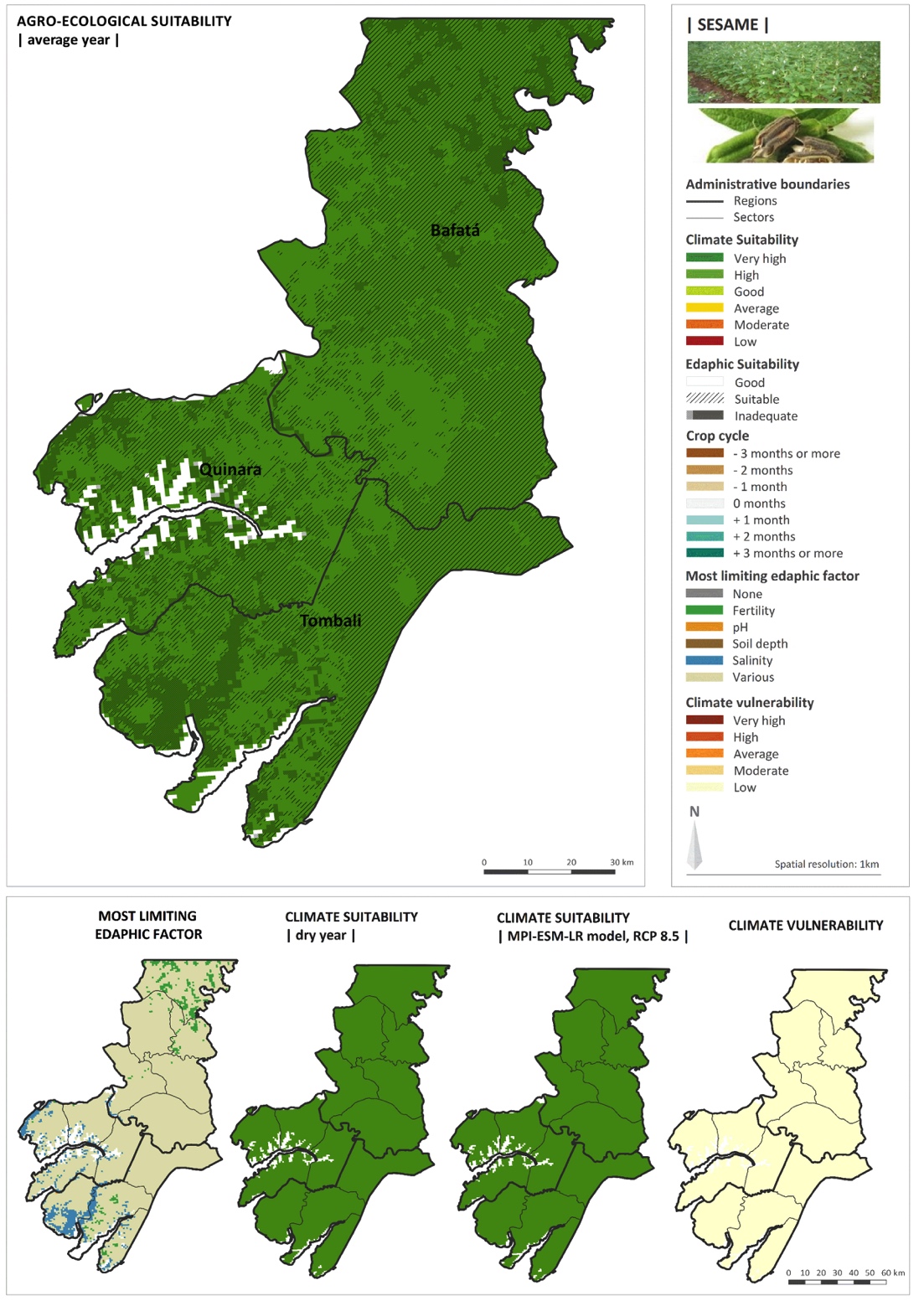
**

| **Class** | **Climatic Parameters** | | | **Edaphic Parameters** | | | |
| --- | --- | --- | --- | --- | --- | --- | --- |
|  | **Temperature (ºC)** | **Precipitation (mm)** | **Crop cycle (days – months)** | **Fertility (% organic carbon)** | **Soil Depth (cm)** | **pH** | **Salinity/**  **Conductiv-ity (dS/m)** |
| Optimal | 20 – 30 | 500 – 1000 | 70 - 3 | > 5 | > 100 | 5.5 – 7.5 | < 2 |
| Suitable | 10 – 20;  30 – 40 | 300 – 500;  1000 – 1500 |  | 1 – 5 | 50 - 100 | 4.5 – 5.5;  7.5 – 8 | 2 – 4 |
| Unsuitable | < 10 ; > 40 | < 300 ; > 1500 |  | < 1 | < 50 | < 4.5 ;  > 8 | > 4 |
